# Quantifying diagnostic intervals and routes to diagnosis for children and young people with cancer in the UK (Childhood Cancer Diagnosis study, CCD): a population-based observational study

**DOI:** 10.1016/j.lanepe.2025.101329

**Published:** 2025-05-27

**Authors:** Dhurgshaarna Shanmugavadivel, Jo-Fen Liu, Timothy A. Ritzmann, Ashley Ball-Gamble, Angela Polanco, Neil Ranasinghe, Kavita Vedhara, Shalini Ojha, David Walker

**Affiliations:** a Lifespan and Population Health, School of Medicine, University of Nottingham, Nottingham, UK; b CCLG: The Children & Young People’s Cancer Association, Leicester, UK; c Children’s Brain Tumour Research Centre, School of Medicine, University of Nottingham, Nottingham, UK; d School of Psychology, Cardiff University, UK & School of Medicine, Keele University, UK

**Keywords:** Childhood cancer, Paediatric oncology, Global health, Public health

## Abstract

**Background:**

Childhood cancer is a global disease burden, with early diagnosis a priority. We quantified diagnostic intervals and referral routes for children and young people (CYP 0–18 years) diagnosed with cancer in the UK.

**Methods:**

All CYP diagnosed between September 2020–March 2023 were eligible. Demographic, referral, and symptom data were collected prospectively. Patient interval (PI), diagnostic interval (DI), and total diagnostic interval (TDI) were calculated.

**Findings:**

1957 CYP (mean age 7.4 years, 55% male, 78% white) participated. Median PI, DI, and TDI were 1.1 (IQR 0.1–4.0; range 0–164), 1.7 (IQR 0.4–5.9; range 0–310), and 4.6 weeks (IQR 2.0–11.4; range 0–310), respectively. Intervals were unaffected by sex, ethnicity or deprivation index (IMD). Median TDI was longest in 15–18 years (8.7 weeks, IQR 3.0–17.4) and bone tumours (12.6 weeks, IQR 6.6–23.4) and shortest in under ones (3.7 weeks, IQR 1.0–8.1) and renal tumours (2.3 weeks, IQR 0.9–5.0). 74% (n = 1438) had 1–3 pre-diagnostic healthcare contacts; 67% (n = 1312) presented emergently, with a median of 4.0 (range 0–26) symptoms. CYP with Langerhans Cell Histiocytosis were most likely to have ≥4 visits when compared with leukaemia (adjusted OR 7.48, 95% CI 3.54–15.82), followed by central nervous system, bone, and soft tissue tumours.

**Interpretation:**

This study highlights equal access to diagnosis for sex, ethnicity and IMD, but disparities for age and diagnostic groups. These data will inform professional and public health strategies and health policy to accelerate diagnosis for all.

**Funding:**

National Institute for Health and Social Care Research (NIHR) DRF-2018-11-ST2-055.

**Research in context**

**Evidence before this study**

We searched EMBASE and MEDLINE for published papers up to January 9, 2025, without language restrictions, using the terms (“time to diagnosis” OR “diagnostic interval$” OR “symptom interval$” AND “child$ adj 5 cancer”).

We found 24 publications studying all childhood cancer types in all age groups, of which 1 was the protocol paper of this study. Of those studying time to diagnosis, the majority were single centre retrospective studies spanning a range of countries including India, France, Cameroon, Scotland, Nigeria and Peru with one danish nationwide population study (Ahrensberg et al., 2013) looking specifically at the diagnostic interval from first primary care contact.

The latest comprehensive systematic review (Lethaby et al., 2013) included 32 papers (10,866 patients) in 2012. They found that time to diagnosis differed by tumour type, with some studies also highlighting an association with older age. The majority of data in the studies were collected retrospectively at single institutions and included an age range of up to 30 and were limited by language bias. A key conclusion was a need for standardised reporting of summary data and terminology of diagnostic intervals and delay in the literature. We could not find evidence of any prospective study where diagnostic intervals and routes to diagnosis of all childhood cancers were recorded. This study bridges these gaps in the literature.

**Added value of this study**

This is the first national multi-centre prospective observational study of diagnostic intervals and routes to diagnosis of CYP with cancer and leukaemia including all UK Principal Treatment Centres (England, Wales, Scotland and Northern Ireland). We report that diagnostic intervals range from the same day to 6 years and are significantly affected by age and diagnosis, however, not affected by sex, ethnicity and deprivation levels. Among teenagers, bone tumours, LCH and rare carcinoma categories standout as having the slowest access to diagnosis, followed by other solid tumours and CNS tumours. At diagnosis, three-quarters have had 1–3 consultations with a healthcare professional and present with a median of 4 symptoms. The overwhelming majority of CYP in the UK present to their GP and an emergency doctor prior to receiving their diagnosis, only 5% are diagnosed by a paediatrician.

**Implications of all the available evidence**

These data will be used to focus efforts on accelerating diagnosis for subgroups with lengthy intervals by providing gold-standard clinical guidance which will inform a new campaign called Child Cancer Smart to raise public and professional awareness. Our infographics of symptom clusters by age, anatomy and diagnostic subgroup can be shared globally. Furthermore, the recent announcement of a new national cancer plan for England with specific paediatric focus is a first, and these data will be used to inform health policy as benchmarks for timely diagnosis and to strengthen referral routes to improve outcomes for all.

## Introduction

Childhood cancer is a global health challenge, affecting 400,000 children and young people (CYP) each year.^1^ Prompt diagnosis is crucial for optimising survival and outcomes. The World Health Organisation (WHO) has prioritised early diagnosis through the Global Initiative for Childhood Cancer (GICC) launched in 2018, with the goal, to offer 2/3rd of children the chance of cure by 2030 and, in doing so, save an additional 1 million children’s lives.^2^ They urged all countries to evaluate and make change, however recent data highlighted the lack of paediatric-specific National Cancer Control Plans (NCCPs) across Europe, with only 4 out of 41 countries having comprehensive paediatric oncology content.^3^

Currently, symptom recognition remains the main route to early detection.^4^ CYP presenting clinically with cancer can have life threatening risks requiring urgent intervention to enable accurate diagnosis, staging, and planning of treatment. Prolonged diagnostic intervals risk sudden death, worsening disability and up-staging.^4^ The consequent need for higher risk therapies affect chances of survival and life-long late effects.^5^

Factors within health systems, many of which are economically and politically driven, determine speed of access to diagnosis and treatment.^6^, ^7^, ^8^ Public and professional awareness, accessibility to health care and access to diagnostics and treatments all affect time to diagnosis. In the UK, prioritisation of cancer screening and public awareness for adults with cancer have become established and contributed to improving outcomes.^9^ However, the level of public awareness of children’s cancer is informed by adult cancer symptom awareness.^10^ Health-seeking behaviours for CYPs are different to adults, with higher use of urgent care services which could impact routes to diagnosis.^11^ Furthermore, within primary care, access to appropriate diagnostic tests often requires referral to children’s secondary or tertiary care which contributes to delays.

In the UK, the award-winning HeadSmart public and professional symptom awareness campaign was associated with halving the time to diagnosis for childhood brain tumours nationally from a median of 14.4 weeks–6.5 weeks, demonstrating that the use of awareness can be effective.^12^ The HeadSmart campaign has been used as a model globally in both LMIC and HIC, encouraging investigation and action, highlighting the importance of sharing evidence-based practice to improve outcomes worldwide.^13^, ^14^, ^15^, ^16^

Currently our understanding of the diagnostic intervals and referral pathways for CYP with cancer in the UK is poor. This information is critical to benchmark progress, identify inequalities and develop professional and public health strategies in order to improve cancer detection and treatment outcomes in childhood. This population-based observational study aims to quantify diagnostic intervals and routes to diagnosis for CYP across the UK.

## Methods

### Study design

Childhood cancer care in the UK is centralised and provided by Principal Treatment Centres (PTC) for Paediatric Oncology and Haematology. This prospective observational study included all 20 PTCs in the UK (Supplementary Fig. S1). Recruitment was open from 31 September 2020 to 31 March 2023, extended from a planned 2-year period due to the COVID-19 pandemic. The study received ethical approval from York and Humber, Leeds West REC (19/YH/0416), and a detailed study protocol has been published.^17^

### Participants

Inclusion criteria were CYP aged 0–18 years with a new diagnosis of cancer and under the care of the PTC during the study period; patients diagnosed outside the UK were not eligible. CYP were identified and recruited by a member of the clinical care team during the first consultation once the initial cancer diagnosis was made. Informed consent was obtained from parents or guardians for participation in the study (Supplementary Table S1).

### Procedure

Data were collected from parents/carers on standardised case report forms by the clinical care team at the first consultation (Supplementary Table S2). Collected data included demographics, Index of Multiple Deprivation (IMD)^18^ (calculated from home postcode), dates of symptom onset, first presentation and diagnosis, route to diagnosis and clinical symptoms at diagnosis. Tumour diagnoses were coded according to the International Classification of Childhood Cancer (ICCC-3),^19^ tumour stage was also recorded (Supplementary Table S3).

The primary outcome was the Total Diagnostic Interval (TDI), defined as time between symptom onset and diagnosis. The secondary outcome measures are the Patient Interval (PI), calculated as the interval between symptom onset and first presentation to healthcare, and the Diagnostic Interval (DI), time between first presentation and diagnosis.^20^ These results are presented as median, interquartile range (IQR), and range for the whole cohort and subgroups.

### Statistical analysis

Descriptive analyses were used to characterise the study population. Sub-analyses by age, sex, ethnicity, IMD, geographical region, and cancer type were performed. Chi-squared or Kruskal–Wallis were used for comparison between groups as appropriate and Bonferroni correction was used for post-hoc pairwise comparisons. The associations between diagnostic delay and potential risk factors (age, sex, ethnicity, IMD, and diagnosis) were explored. Adjusted odds ratios (adjORs) and 95% confidence intervals were estimated using multivariate logistic regression. All analyses were conducted using SPSS (IBM SPSS Statistics, Version 29.0.2.0 NY: IBM Corp). A p-value < 0.05 was considered statistically significant.

### Role of the funding source

The funder of the study had no role in study design, data collection, data analysis, data interpretation, or writing of the report.

## Results

A total of 1957 participants were included in the analysis (Table 1) representing 53% of incident cases reported by PTCs during the study period (Supplementary Table S3). The most common diagnoses were leukaemia (778, 40%), CNS tumours (275, 14%) and lymphoma (254, 13%).

**Table 1:** Summary characteristics of cohort (n = 1957).

	n	Col %
**Sex**		
Male: Female	1075:882	55%:45%
**Region**		
England	1690	86%
Wales	5	0.3%
Scotland	211	11%
Northern Ireland	51	3%
**Ethnicity**		
White	1528	78%
Mixed/Multiple ethnic groups	91	5%
Asian/Asian British	156	8%
Black/African/Caribbean/Black British	43	2%
Other ethnic group	56	3%
Not known	83	4%
**IMD in quintile**^a^		
1 (most deprived)	391	21%
2	341	19%
3	341	19%
4	392	21%
5 (least deprived)	369	20%
**Age group**		
Under 1	152	8%
1–4	717	37%
5–9	432	22%
10–14	409	21%
15+	247	13%
**Diagnosis**		
Leukaemia	778	40%
Lymphoma & related	254	13%
CNS tumour	275	14%
Neuroblastoma	105	5%
Retinoblastoma	32	2%
Renal tumour	139	7%
Hepatic tumour	40	2%
Bone tumour	124	6%
Soft tissue sarcoma	128	7%
Germ cell tumour	28	1%
Carcinoma & melanoma	13	0.7%
Other & unspecified malignant	5	0.3%
Langerhans Cell histiocytosis (LCH) and other histiocytosis	36	2%

a IMD data only available for 1834 CYP.

Table 2 summarises the key features of patients’ route to diagnosis. About three-quarters (1438, 74%) of CYP had 1–3 HCP visits before diagnosis. Two-thirds (1312, 67%) were diagnosed via emergency presentation, including attendance at the emergency department, emergency referral, transfer, or admission, and for 43 (2%) CYP, the cancer was an incidental finding.

**Table 2:** Route to diagnosis pathways.

	n	%
**The first HCP patient saw about symptoms**		
GP	1113	57%
Emergency doctor	562	29%
Paediatrician	107	5%
Optometrist	35	2%
Sub-specialist doctor	35	2%
WIC/UCC/MIU	16	0.8%
NHS111/NHS24^a^	14	0.7%
Nurse practitioner	13	0.7%
Pre/peri-natal	12	0.6%
Dentist	10	0.5%
Health visitor	10	0.5%
Physiotherapist	6	0.3%
Private	5	0.3%
Pharmacist	3	0.2%
School nurse	2	0.1%
Other	8	0.4%
**Number of HCP visits before diagnosis**		
1–3	1438	74%
4–6	375	19%
7–9	88	5%
10+	51	3%
**Source of referral leading to diagnosis**		
Emergency presentation^c^	1312	67%
GP referral	463	24%
Other^b^	178	9%
Unknown	3	0.2%
**Incidental finding**		
No	1913	98
Yes		
Asymptomatic	40	2
Antenatal diagnosis	3	0.2
**Place of care which requested investigation identifying the cancer**		
Inpatient	784	40%
Emergency	572	29%
Outpatient	366	19%
Primary care	208	11%
Private	11	0.6%
Other	10	0.5%
Not known	5	0.3%

WIC = Walk-in-centre; UCC = Urgent Care Centre; MIU = Minor Injury Unit are urgent in-person medical or injury advice.

a NHS111 (England & Wales) NHS 24 (Scotland) are telephone helplines for non-emergency but urgent medical advice.

b Diagnosed by another specialty (n = 131), active surveillance (n = 33), private (n = 11) and other referral pathway not specified (n = 3).

c Emergency presentation: an emergency route via A&E, including emergency GP referral, emergency consultant outpatient referral, emergency transfer, emergency admission or attendance.

### Symptoms at diagnosis

**Fig. 1:**
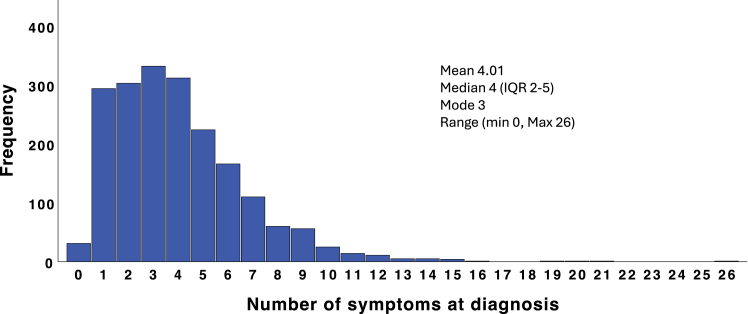
**Distribution of number of symptoms at diagnosis**.

**Fig. 2:**
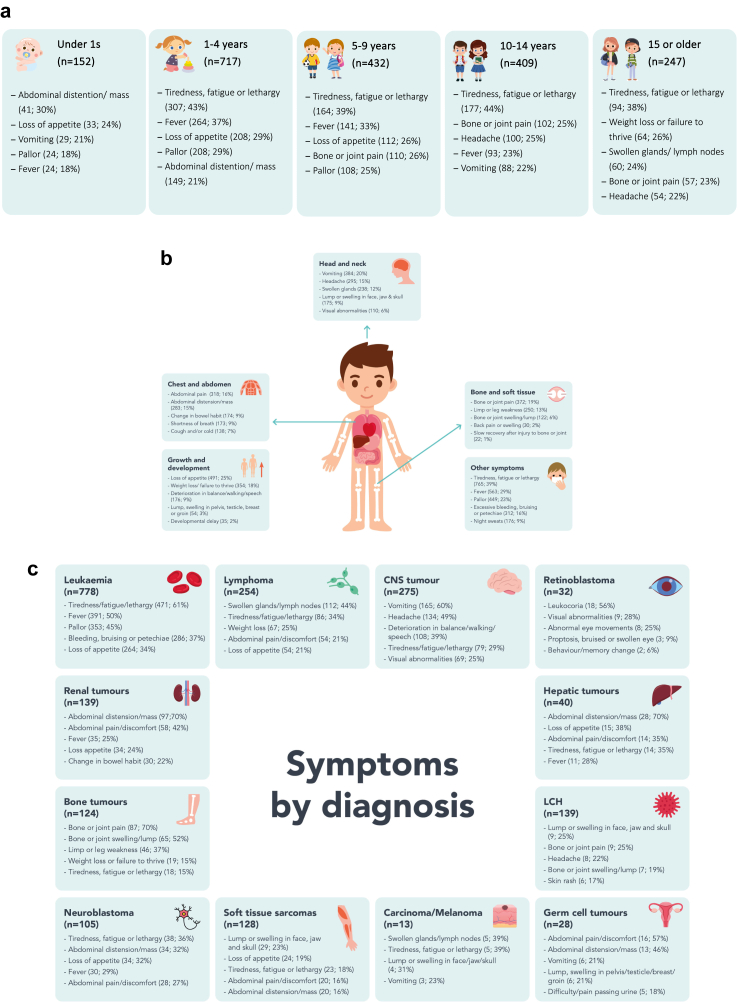
**The five most commonly reported symptoms (a) by age group (b) by anatomical region and (c) by tumour type**.

The number of symptoms at presentation ranged from none to 26 with a median of 4 (IQR 2–5) symptoms. Approximately half (960/1957; 49%) of the CYP presented with 3 or fewer symptoms, whilst 295/1957 (15%) presented with 7 or more symptoms (Fig. 1). A total of 63 different symptoms were reported, with the most frequent being tiredness/fatigue (765; 39%), fever (563; 29%), loss of appetite (491; 25%), pallor (449; 23%) and vomiting (348; 20%). Symptoms stratified by age, anatomical region and tumour type are shown in Fig. 2.

### Diagnostic intervals

**Fig. 3:**
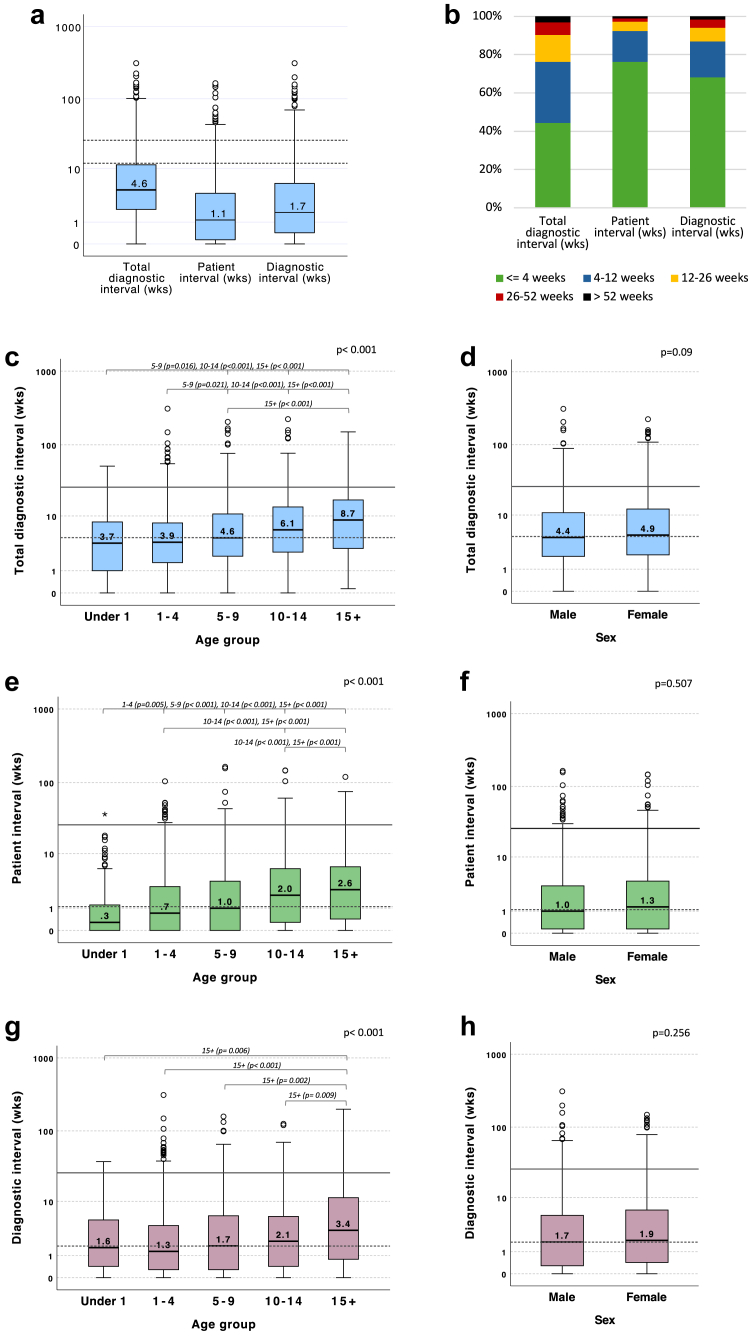
**Total diagnostic interval (TDI), patient interval (PI) and diagnostic interval (DI) in weeks.** (a) Box plots. (b) The proportions of patients with intervals of ≤4 week, 4–12 week, 12–26 weeks, 26–52 weeks and >52 weeks. Details see Supplementary Table S2. (c–h) Box plots by age group and sex. In all box plots, dashed lines represent the group median (TDI 4.6 weeks, PI 1.1 weeks, DI 1.7 weeks); solid lines represent 26 weeks, respectively.

CYP with two or more key dates missing (n = 19) or illogical date sequence (n = 5) were excluded, leaving 1933 CYP included in the analysis (Supplementary Fig. S2). The median (IQR, range) diagnostic intervals were TDI 4.6 weeks (2.0–11.4, 0–310); PI 1.1 weeks (0.1–4.0, 0–164); and DI 1.7 weeks (0.4–5.9,0–310) (Fig. 3, Supplementary Table S4). About 10%, 3%, and 6% of CYP had TDI, PI, and DI over 26 weeks, respectively.

### Intervals by demographic

Intervals by demographics age, sex, ethnicity, IMD and region are shown in Fig. 3, Supplementary Figs. S3 and S4 and Supplementary Table S5.

There was no difference in intervals by sex, ethnicity or IMD. The distribution of intervals differed across age groups (Fig. 3, Supplementary Fig. S3 and Supplementary Table S5). The longest median TDI was in the 15+ group (8.7 weeks; IQR 3.0–17.4), and the shortest in the under 1 group (3.7 weeks; IQR 1.0–8.1). For PI, young people aged 15+ also had the longest interval (2.6 weeks; IQR 0.4–6.3), while the under 1 group had the shortest (0.3 weeks; IQR 0.0–1.2). The longest median DI was again in the 15+ group (3.4 weeks; IQR 0.8–11.4), and the shortest in the 1–4 years group (1.3 weeks; IQR 0.3–4.1).

Regional differences were also observed in PI and DI (Supplementary Fig. S4 and Supplementary Table S5). The PI was shorter in England and Wales (median 1.0 weeks; IQR 0.1–4.0) compared to Scotland (2.0 weeks; IQR 0.3–4.4; p = 0.021). Conversely, the DI was longer in England and Wales, with a median of 1.9 weeks (IQR 0.4–6.0) compared to 1.0 weeks (IQR 0.1–4.0) in Scotland (p < 0.001).

### Intervals by diagnosis

**Fig. 4:**
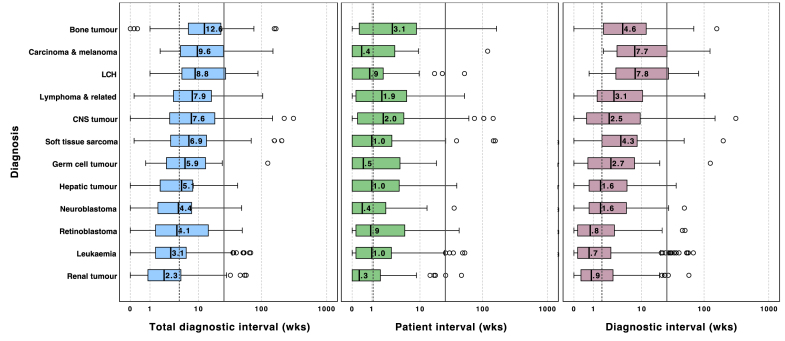
**Total diagnostic interval (TDI), patient interval (PI) and diagnostic interval (DI) in weeks by diagnosis.***Diagnoses are ranked in descending order of median TDI*. Dashed lines represent the group median (TDI 4.6 weeks, PI 1.1 weeks, DI 1.7 weeks); solid line represents 26 weeks, respectively. Subcategory with n < 10 (other & unspecified, n = 5) is not shown.

Variation of diagnostic intervals are shown in Fig. 4 and Supplementary Table S5. The diagnoses with short median TDIs were renal tumours (2.3 weeks; IQR 0.9–5.0), leukaemia (3.1 weeks; IQR 1.4–6.1), and retinoblastoma (4.1 weeks; IQR 0–50.4). The longest median TDI was observed in bone tumours (12.6 weeks; IQR 6.6–23.4), followed by carcinoma/melanomas (9.6 weeks; IQR 4.9–25.6) and LCH (8.8 weeks, IQR 5.1–27.6).

The diagnoses with the longest median PI were bone tumours (3.1 weeks; IQR, 0.3 to 8.7), CNS tumours (2.0 weeks; IQR, 0.2 to 5.2) and lymphoma (1.9; IQR 0.1–5.9). The diagnoses with the longest median DI were LCH (7.8 weeks; IQR 3.4–27.6) and carcinoma/melanomas (7.7 weeks; IQR 3.6–26.6) and bone tumours (4.6 weeks; IQR 1.9–12.0).

Among diagnoses with high TDI, bone tumours also showed high median PI (3.1 weeks; IQR 0.3–8.7) and DI (4.6 weeks; IQR 1.9–12.0). Carcinoma/melanomas and LCH, on the other hand, had high median DI (7.7 weeks; IQR 3.6–26.6 and 7.8 weeks; IQR 3.4–27.6, respectively) and relatively low median PI (0.4 weeks; IQR 0.0–3.6 and 0.9 weeks; IQR 0.0–2.0, respectively). Other diagnoses with high median PI and DI included CNS tumours (PI 2.0 weeks; IQR 0.2–5.2; DI 2.5 weeks; IQR 0.6–9.5) and lymphoma (PI 1.9 weeks; IQR 0.1–5.9; DI 3.1 weeks; IQR 1.3–10.4).

### Intervals by route to diagnosis

**Fig. 5:**
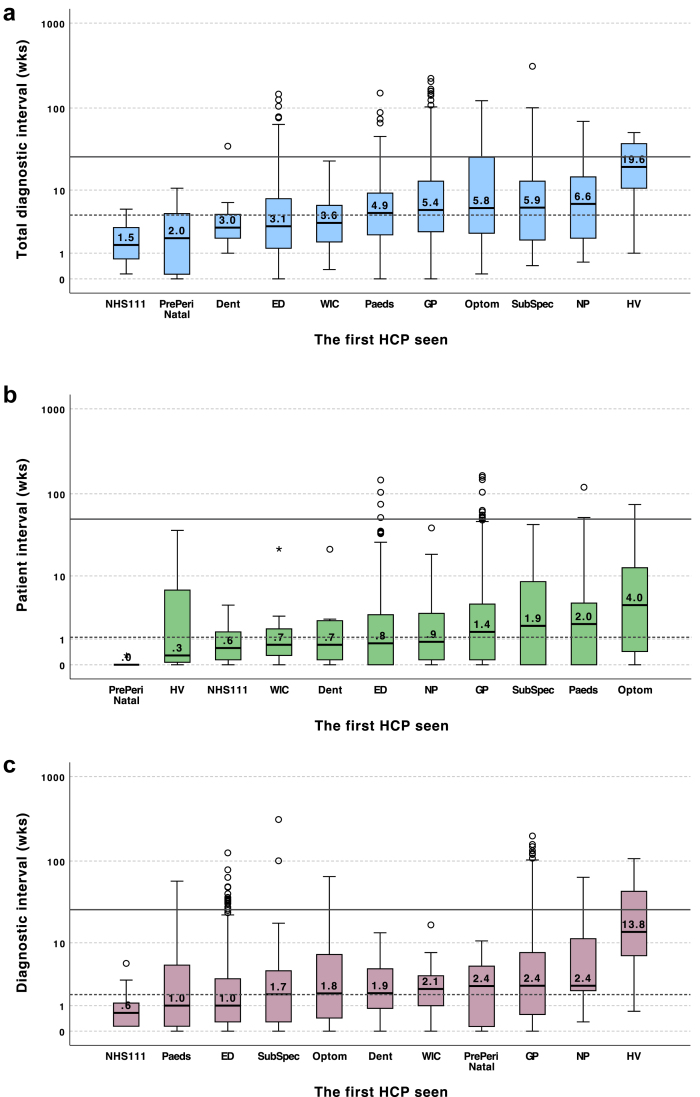
**Box plots showing(a)total diagnostic interval (TDI),(b)patient interval (PI) and(c)diagnostic interval (DI) in weeks by first healthcare professional seen.** Results are ranked in ascending order. *Dashed lines represent the group median (PI 1.1 weeks, DI 1.7 weeks, TDI 4.6 weeks); solid line represents 26 weeks, respectively.* Abbreviations: **ED:** Emergency doctor, **Paeds:** Paediatrician, **Dent:** Dentist, **Optom:** Optometrist, **NP:** Nurse practitioner, **HV**: Health visitor, **NHS111**: NHS111/NHS24, **WIC:** Walk-in Centre/Urgent Care Centre/Minor Injuries Unit, **SubSpec**: Sub-specialist doctor, **PrePeriNatal**: Pre/peri-natal. Subcategories with n < 10 (pharmacist n = 3, school nurse n = 2, private n = 5, physiotherapist n = 6 and other n = 8) are not shown.

Variation by the first healthcare professional (HCP) seen is shown in Fig. 5 and Supplementary Table S6. The most common healthcare professionals seen at initial presentation were GPs (median TDI 5.4 weeks, IQR 2.6–13.0, emergency department doctors (3.1 weeks, IQR 1.3–7.7) or paediatricians (4.9 weeks; IQR 2.32–9.1).

Intervals by number of HCP contacts and place of care when the diagnostic test was requested are shown in Supplementary Fig. S5 and Supplementary Table S6. As the number of contacts increased, the intervals increased. Patients diagnosed via emergency department had shorter median TDI and DI compared to those under the care of inpatient or outpatient. Intervals by source of referral are shown in Supplementary Fig. S6 and Supplementary Table S6, with emergency referrals, as expected, having the shortest median diagnostic intervals.

### Exploring lengthy intervals

In the absence of an agreed definition, we defined delayed diagnosis as a TDI in the top quartile (≥11.5 weeks), a TDI of 6 months or longer, and four or more visits prior to diagnosis.

Age was a significant risk factor for longer TDI. Compared to children under 1, those aged 10–14 years and 15+ were more likely to have TDI in the top quartile, independent of sex, ethnicity, IMD and diagnosis (adj OR 1.89 95% CI 1.07–3.32 and 3.58 95% CI 1.95–6.57, respectively). Diagnostic subgroup also showed significant associations with potential delay. Nine out of the 12 diagnoses with more than 10 cases reached significant level with adjusted ORs ranged from 2.34 to 6.86 (Table 3 and Supplementary Table S7). Diagnoses with an adj OR above 5 included LCH (adj OR 6.86, 95% CI 3.20–14.69), bone tumours (adj OR 6.36, 95% CI 3.99–10.12) and CNS tumours (adj OR 5.24, 95% CI 3.69–7.46).

**Table 3:** Factors associated with long total diagnostic interval and multiple visits (4 times or more) before diagnosis.

	Total diagnostic interval (weeks)^a^			Number of HCP visits before diagnosis^b^	
	n (%)	Q4: 11.5+ wks	>6 months (26 wks)	n (%)	4+ visits
		Adj OR (95%CI)^c^	Adj OR (95%CI)^c^		Adj OR (95%CI)^c^
**Age group**		*p* < *0.001*	*p = 0.079*		*p = 0.690*
Under 1	142 (8%)	1.00	1.00	151 (8%)	1.00
1–4	685 (37%)	1.22 (0.73–2.06)	1.80 (0.75–4.30)	716 (37%)	1.28 (0.81–2.02)
5–9	414 (22%)	1.73 (0.99–3.01)	2.76 (1.11–6.86)	431 (22%)	1.26 (0.77–2.08)
10–14	395 (21%)	1.89 (1.07–3.32)	2.48 (0.98–6.25)	408 (21%)	1.11 (0.66–1.85)
15+	239 (13%)	3.58 (1.95–6.57)	3.33 (1.26–8.77)	246 (13%)	1.35 (0.77–2.37)
**Sex**		*p = 0.151*	*p = 0.202*		*p = 0.836*
Male	1034 (55%)	1.00	1.00	1074 (55%)	1.00
Female	841 (45%)	1.19 (0.94–1.52)	1.25 (0.89–1.75)	878 (45%)	1.02 (0.82–1.28)
**Ethnicity**		*p = 0.733*	*p = 0.695*		*p = 0.966*
White	1462 (81%)	1.00	1.00	1523 (81%)	1.00
Other ethnic group	335 (19%)	0.95 (0.69–1.29)	0.91 (0.58–1.43)	346 (19%)	0.99 (0.75–1.32)
**IMD in quintile**		*p = 0.170*	*p = 0.455*		*p = 0.508*
1 Most deprived	374 (21%)	1.00	1.00	390 (21%)	1.00
2	329 (19%)	0.70 (0.48–1.04)	0.64 (0.37–1.10)	341 (19%)	0.91 (0.64–1.29)
3	319 (18%)	0.84 (0.58–1.24)	0.74 (0.43–1.27)	341 (19%)	0.95 (0.67–1.35)
4	379 (22%)	0.90 (0.62–1.29)	0.69 (0.41–1.16)	390 (21%)	1.19 (0.85–1.66)
5 Least deprived	354 (20%)	1.12 (0.78–1.61)	0.87 (0.53–1.44)	368 (20%)	0.92 (0.65–1.30)
**Diagnosis**		*p* < *0.001*	*p* < *0.001*		*p* < *0.001*
Leukaemia	749 (40%)	1.00	1.00	776 (40%)	1.00
Lymphoma & related	244 (13%)	3.45 (2.34–5.10)	3.92 (2.13–7.24)	253 (13%)	1.97 (1.36–2.85)
CNS tumour	263 (14%)	5.24 (3.69–7.46)	7.85 (4.62–13.33)	274 (14%)	2.69 (1.96–3.70)
Neuroblastoma	99 (5%)	2.34 (1.30–4.22)	1.28 (0.37–4.45)	105 (5%)	1.73 (1.05–2.83)
Retinoblastoma	29 (2%)	3.81 (1.52–9.59)	10.27 (3.39–31.07)	32 (2%)	0.75 (0.25–2.23)
Renal tumour	131 (7%)	1.36 (0.76–2.45)	1.47 (0.54–4.01)	138 (7%)	0.72 (0.42–1.24)
Hepatic tumour	38 (2%)	2.22 (0.92–5.35)	3.55 (0.99–12.80)	40 (2%)	1.38 (0.63–3.01)
Bone tumour	119 (6%)	6.36 (3.99–10.12)	7.09 (3.71–13.55)	124 (6%)	2.58 (1.64–4.04)
Soft tissue sarcoma	124 (7%)	3.79 (2.38–6.04)	3.66 (1.74–7.67)	128 (7%)	2.50 (1.63–3.84)
Germ cell tumour	27 (1%)	3.99 (1.54–10.38)	1.36 (0.17–10.76)	28 (1%)	1.01 (0.33–3.08)
Carcinoma & melanoma	13 (0.7%)	4.36 (1.25–15.23)	4.96 (0.98–25.07)	13 (0.7%)	4.98 (1.48–16.77)
Other & unspecified^d^	5 (0.3%)	–	70.84 (6.02–833.86)	5 (0.3%)	8.59 (0.76–96.66)
LCH	34 (2%)	6.86 (3.20–14.69)	19.22 (8.01–46.10)	36 (2%)	7.48 (3.54–15.82)

a Patients with missing data were not included in the analysis, valid n = 1875.

b Patients with missing data were not included in the analysis, valid n = 1952.

c Adjusted for all factors included in the table. Details see Supplementary Tables S7–S9.

d Less than 10 cases in the group.

A similar pattern was also observed when using TDI ≥ 6 months as the definition of delay (Table 3 and Supplementary Table S8). The risk of longer TDI started to increase from 5 years, with CYP aged 15+ years showing the highest adjusted OR of 3.33 (95% CI 1.26–8.77). Among diagnoses, six of the 12 cancer types were significantly associated with a TDI of ≥6 months (Table 3, adj ORs 3.66–19.22). Subgroups with an odds ratio above 5 were LCH (adj OR 19.22, 95% CI 8.01–46.10), retinoblastoma (adj OR 10.27, 95% CI 3.39–31.07), CNS tumours (adj OR 7.85 95% CI 4.62–13.33) and bone tumour (adj OR 7.09, 95% CI 3.71–13.55).

There was no association between age and having four or more HCP visits before diagnosis. Significant associations were found in seven cancer types (Table 3 and Supplementary Table S9, adj ORs 1.97–7.48). Only LCH showed an OR over 5 (adj OR 7.48, 95% CI 3.54–15.82). Other diagnoses with ORs between 2 and 5 included carcinoma & melanoma (adj OR 4.98, 95% CI 1.48–16.77), CNS tumour (adj 2.69, 95% 1.96–3.70), bone tumour (adj OR 2.58 95% CI 1.64–4.04) and soft tissue sarcoma (adj OR 2.50, 95% CI 1.63–3.84).

There was no association between sex, ethnicity, or IMD and any definition of diagnostic delay in the study population.

## Discussion

Here we present a population-based observational study to measure patient, diagnostic and total diagnostic intervals as well as common routes to diagnosis of childhood cancer in the UK, whilst also identifying evidence of current inequalities. Our central finding was that in the UK, half of all CYP are diagnosed with cancer within 4.6 weeks of first presentation, but for many it takes longer, with the longest taking 310 weeks. Understanding the factors that put CYP at risk of a slower diagnosis is the focus for this discussion, as modifying their experiences offer the greatest opportunity for improving outcomes. Critically, our analysis shows that sex, ethnicity and IMD do not impact on diagnostic intervals, suggesting that the UK health system offers equal access across the childhood population for these characteristics.

The previously reported low levels of awareness of child cancer risks and clinical presentation in the UK, coupled with the WHO’s effort to improve childhood cancer survival rates worldwide to 60% by 2030, has highlighted the need to gather UK-wide data. As shown with HeadSmart,^12^ the provision of nationwide evidence of diagnostic intervals is critical in achieving this ambition, by providing a baseline from which to improve cancer detection and treatment outcomes, as well as inform education and awareness strategies and the focus of future research.

Our finding of equity across sex, ethnicity and IMD differs to UK adult data where female sex, low socioeconomic status and ethnicity has been shown to be associated with longer intervals depending on cancer type.^21^ In contrast, our analysis shows that age, region and diagnostic group was significantly associated with differing intervals.

As age increased, all intervals increased and the gap between mean and median increased, indicating a skewed distribution. This is comparable to the BRIGHTLIGHT data which reported cancer diagnostic timeliness in 12–24 year olds finding a median TDI of 8.9 weeks. However, nearly a decade on, there has been little improvement in intervals, with a median TDI of 8.7 weeks for our 15+ group.^22^ There is, therefore, an urgent need to focus and rethink strategies for accelerating diagnosis for this group given that the risk of long-term depression is doubled if TDI is 2 months or more and the diagnoses they experience are different to younger children.^23^^,^^24^

Geographical region also had an impact; whilst it takes longer for CYP in Scotland to first attend their HCP, once they have been seen their time to diagnosis is shorter than in England. Further research is required to understand these differences, however, structural differences in service organisation and delivery may be among potential causal factors.

Diagnostic intervals also differed by diagnostic subgroup which fits with previously published data.^25^^,^^26^ The embryonal tumours (renal, neuroblastoma, hepatic, retinoblastoma, rhabdomyosarcoma) and leukaemia had the shortest intervals whilst bone tumours, lymphomas, CNS tumours, LCH and “others and unspecified” had the longest intervals. We hypothesise that awareness, visibility of presenting symptoms and access to diagnostic tests is greater in those with shorter intervals. It is important to note that shorter intervals are not always associated with reduced mortality, and can be secondary to aggressively progressing tumours. Nonetheless, previous experience with HeadSmart demonstrated that enhancing public and professional awareness of symptomatology and providing evidence-based guidance for assessment/investigation was associated with halving the TDI nationally.^12^ Similar approaches in bone tumours, lymphoma and LCH may, therefore, also improve outcomes. The diverse range of entities in “other and unspecified” would be more challenging to address and would potentially require different approaches.

A symptom awareness campaign, however, would not be without its challenges. Sixty-three different symptoms were reported overall, confirming the plethora of symptoms with which childhood cancers present; and the most frequent symptoms were non-specific: tiredness, fever, loss of appetite, pallor and vomiting. Categorisation of presenting symptoms by anatomical region confirms childhood cancer as a multi-system, “head to toe” set of diseases which can present to the full range of medical and surgical specialists, some of whom may predominantly treat adults. Stratifying symptomatology by diagnosis has already proven successful in accelerating diagnosis for childhood brain tumours and could be replicated to address diagnoses with skewed interval distributions in this study.^12^

The number of contacts, first HCP seen, and diagnostic route influenced all diagnostic intervals. Children with cancer see their doctors more frequently, especially in the 3 months prior to diagnosis. Three-quarters of this cohort were diagnosed after 3 or fewer visits to a HCP, however almost 1 in 10 had seven or more visits. Dommett et al. identified 12 symptoms which increased the prior probability of childhood cancer from 0.4 in 10,000 to at least 4 in 10,000. When these features were present in a child who attended for the third time in 3 months, their risk of cancer increased further.^27^ This is important to consider in addition to symptom awareness, when providing guidance for the public and healthcare professionals for further investigation in CYP with unexplained symptoms.

For approximately 90%, their first contact was their GP, emergency department or paediatrician. Emergency presentations are recognised as important predictors of cancer outcomes for adults with patients presenting as an emergency having significantly worse outcomes.^28^ In this cohort, just over two-thirds (67%) presented to the emergency department immediately prior to receiving their diagnosis, compared to only 24% reported in the “routes to diagnosis” UK linkage study for adults and children, highlighting a key difference in how CYPs present.^28^ The shortest intervals were associated with initial contact with the emergency department, NHS 111, Walk-In Centres and dentists. A small proportion of these emergency presentations will be CYP who present with an acute or life-threatening presentation warranting prompt investigation. However, for others, easier access to initial investigations when presenting through secondary care may be a reason for shorter intervals through this route. What is yet to be confirmed is whether emergency presentations are ‘protective' for CYP, given that intervals are shorter for this cohort. Our follow up outcome data will address this. The slowest access came through health visitors, nurse practitioners, sub specialists and optometrists seeing children in out-patient clinics, private consultation and other settings. The reasons for longer intervals for each of these groups are not easily discerned from the data. However, some of these groups cannot initiate diagnostic referral or testing independently.

The analysis of lengthy intervals identified age as a significant risk factor with those older than 15, more than 3 times likely to experience lengthy TDIs. Diagnostic subgroup was also a risk factor. LCH, bone tumours and CNS tumours were all at least 5 times more likely to have a TDI in the top quartile or a TDI of 6 months or longer. With regards to having 4+ visits, significant associations were found with diagnosis only; LCH was seven times likely to present 4 or more times prior to diagnosis, carcinoma 5 times more likely, and bone tumours, CNS tumours and soft tissue sarcomas 2.5 times more likely. Interestingly, these findings for bone tumours and sarcomas are similar to pre-referral consultation data in adults.^29^ This suggests that these diagnoses either present with more non-specific presentations or that there is a lack of awareness or both.

Overall, these data could be used to justify a range of benchmarks for a ‘timely diagnosis’ and further support the development of professional and public health strategies to accelerate diagnosis for the subgroups with lengthier intervals.

This study included all PTCs treating CYP with cancer in the UK. Whilst the impact of the COVID-19 pandemic affected projected recruitment, this is the first and largest known study looking at diagnostic intervals and referral pathways globally. We have previously reported on the impact of COVID upon childhood cancer diagnosis and found no significant differences in TDI with a pre-COVID cohort.^30^ Follow-up data collection at 5 years post-diagnosis will commence in September 2025. This will enable analyses of associations between diagnostic intervals and refractory disease, relapse and survival providing additional insight into whether diagnostic intervals affect outcomes, which is currently unknown.

We acknowledge the possibility of recall bias in this study, as initial symptom onset dates were obtained from CYP and their families at diagnosis. This was minimised by ensuring data were collected at first presentation during routine history taking prior to introducing the study however random errors could have occurred where parental/patient recall of symptoms may have been ascribed incorrectly to cancer. Another limitation is the lack of data on non-participation which does not allow comparison of characteristics with non-participants, however when compared against nationally reported data, this cohort is largely representative of annual cancer incidence and statistics (Supplementary Table S10) aside from an under-representation of young people (15–18).^31^ Despite this, we have shown a statistically significant difference in intervals in this group and our results are comparable to data from the BRIGHTLIGHT study which has studied this age group in detail.^22^

### Conclusion

This study is the first to our knowledge to document diagnostic intervals and routes to diagnosis in a population cohort in the UK, and internationally. The majority of CYP present as an emergency, with 3 or more symptoms, and were diagnosed within 3 or less visits. However, half of CYP diagnosed with cancer in the UK are taking longer than 4 weeks to be diagnosed. Age and diagnosis are significant risk factors for lengthier intervals, with young people and bone tumours requiring urgent focus. There were no major differences across ethnicity, sex or IMD when assessing lengthier diagnostic intervals. In order to learn from our key findings, we propose that these data can be used to inform professional and public health strategies and health policy to accelerate diagnosis and improve outcomes.

## Contributors

DS, JFL, TR, AP, ABG, DAW, SO, KV designed the study. DS JFL and SO accessed and verified the dataset. DS and JFL conducted the statistical analysis with clinical guidance from TR and DAW. DS wrote the first draft of the report with input from JFL, SO, KV, ABG, AP, NR, TR and DAW. All authors had full access to all data in the study and had final responsibility for the decision to submit for publication.

## Data sharing statement

De-identified data will be provided by the authors upon reasonable request, pending scientific review and completion of a data transfer agreement. Requests should be directed to the corresponding author.

## Editor note

The Lancet Group takes a neutral position with respect to territorial claims in published maps and institutional affiliations.

## Declaration of interests

ABG is the CEO of the Children’s Cancer and Leukaemia Group. DAW is the CEO of DAW MRL Consultancy Ltd, providing medico-legal expertise on childhood cancer care. DS has been appointed as Vice-Chair of the Children’s and Young Persons Cancer Taskforce at the Department of Health, an unpaid role commencing in March 2025. DAW MRL Consultancy and DS’s roles had no influence on the study design, data collection, analysis, or report preparation. The remaining authors declare no competing interests.
