# Supplementary material for: Quantifying diagnostic intervals and routes to diagnosis for children and young people with cancer in the UK (Childhood Cancer Diagnosis study, CCD): a population-based observational study
Source: Lancet Reg Health Eur. 2025 May 27;54:101329. doi: 10.1016/j.lanepe.2025.101329 (PMC12266182; doi:10.1016/j.lanepe.2025.101329)
Supplement: Supplementary Figure S1 [file mmc1.pdf]

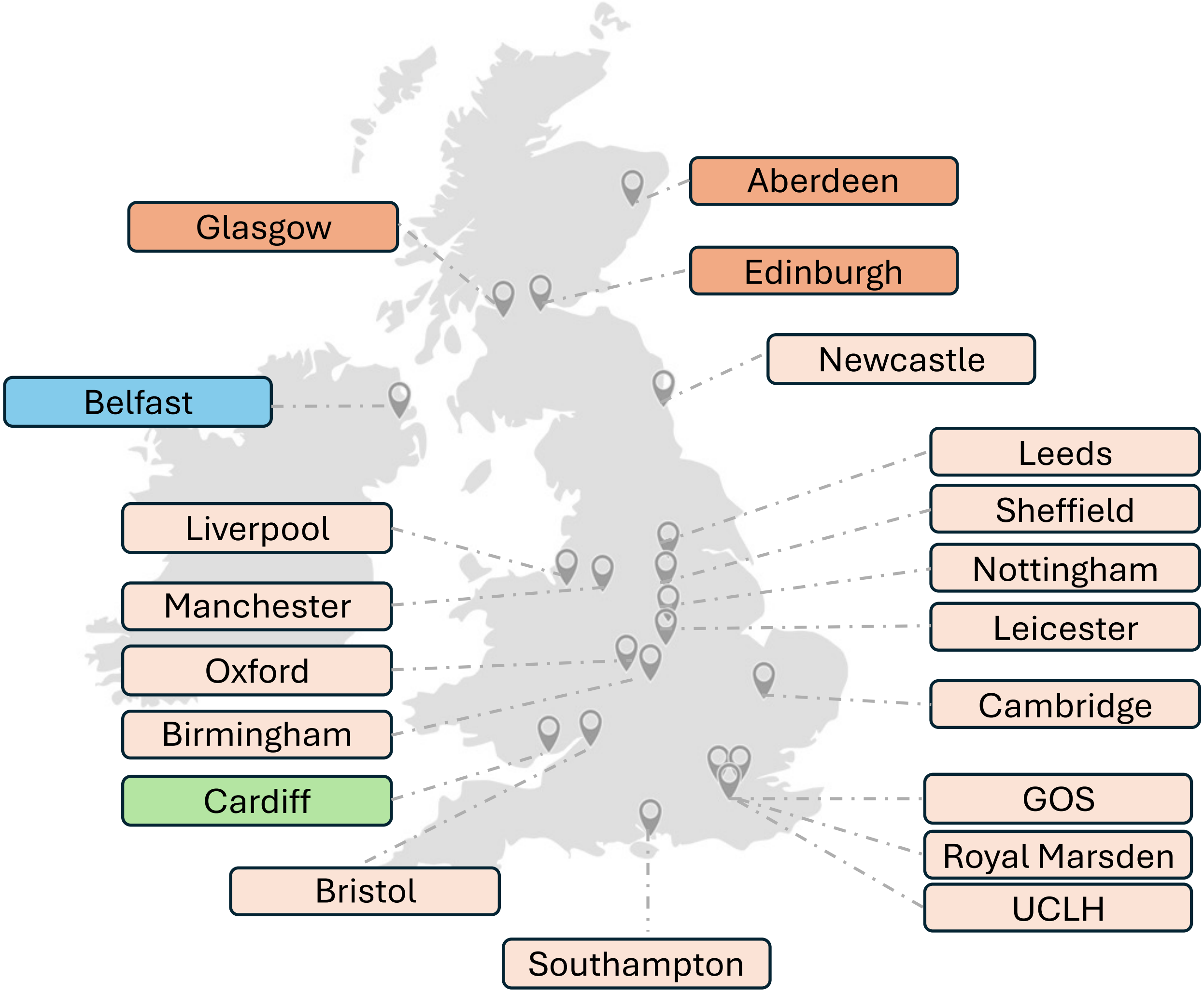

Figure S1: A map of the Principal Treatment Centres (PTCs) across the UK.
(Key: dark orange= Scotland, blue= Northern Ireland, green = Wales, light orange = England).
