# Supplementary figures and images for: Quantifying diagnostic intervals and routes to diagnosis for children and young people with cancer in the UK (Childhood Cancer Diagnosis study, CCD): a population-based observational study
Source: Lancet Reg Health Eur. 2025 May 27;54:101329. doi: 10.1016/j.lanepe.2025.101329 (PMC12266182; doi:10.1016/j.lanepe.2025.101329)

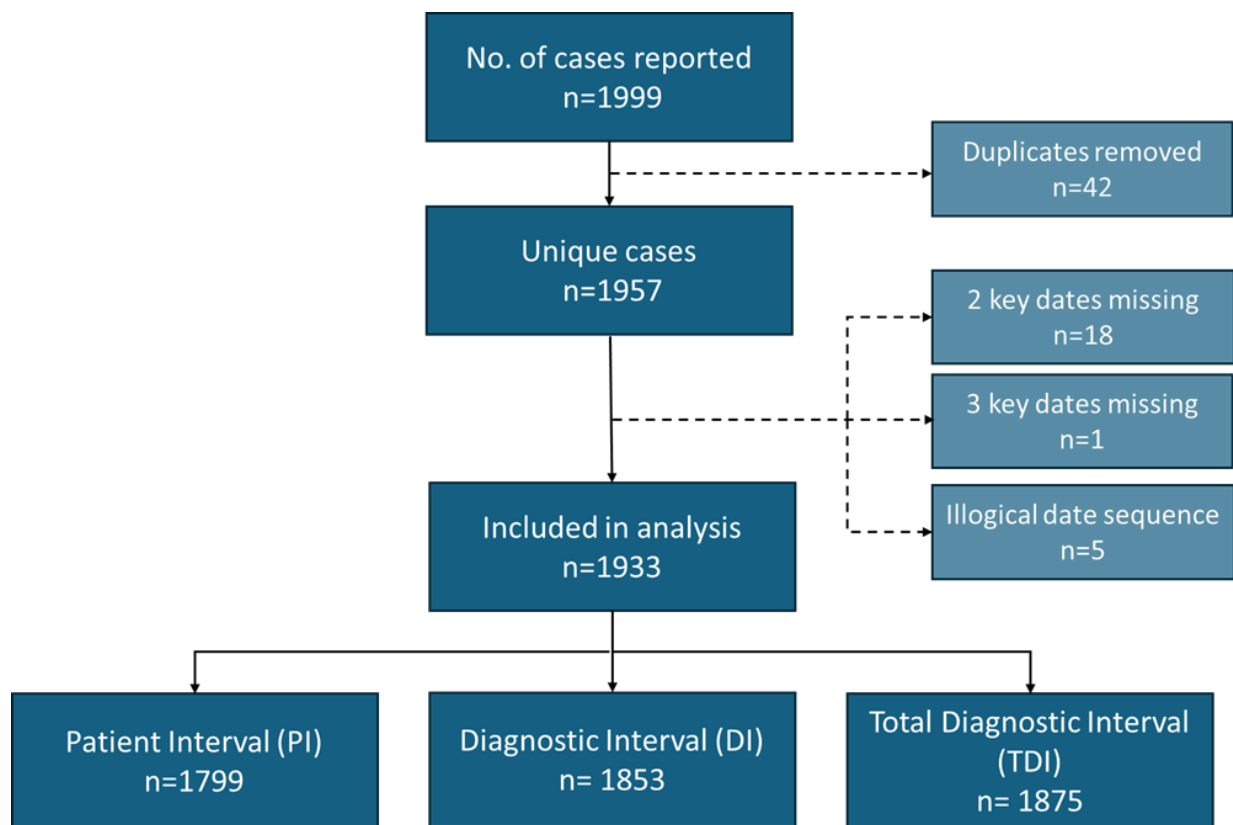

**Figure S2:** Number of cases included in the interval analysis

Supplement: Supplementary Figure S2 [file mmc2.pdf]
