# Supplementary material for: Quantifying diagnostic intervals and routes to diagnosis for children and young people with cancer in the UK (Childhood Cancer Diagnosis study, CCD): a population-based observational study
Source: Lancet Reg Health Eur. 2025 May 27;54:101329. doi: 10.1016/j.lanepe.2025.101329 (PMC12266182; doi:10.1016/j.lanepe.2025.101329)
Supplement: Supplementary Figure S3 [file mmc3.pdf]

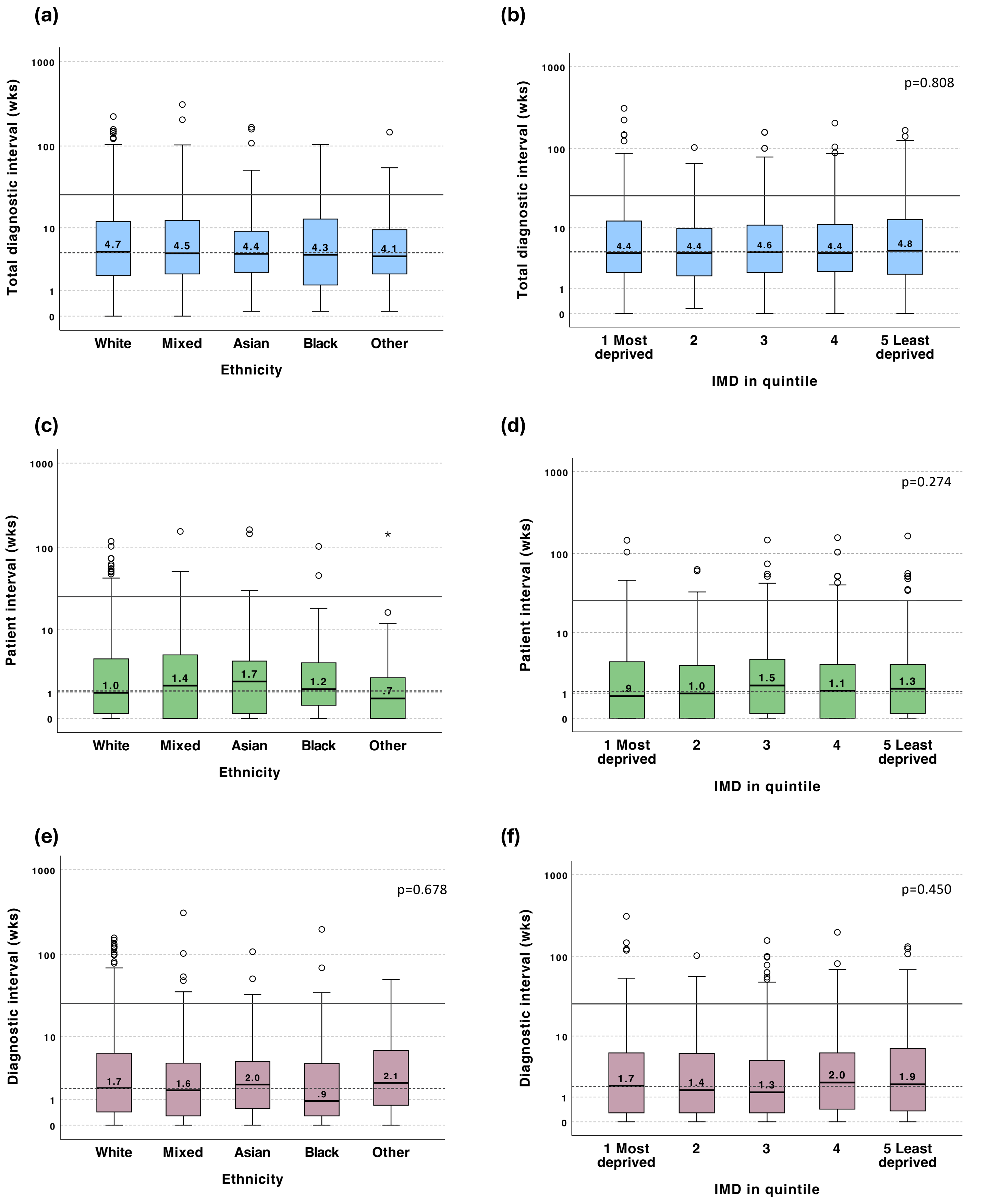

Figure S3 Box plots showing (a-b) total diagnostic interval (TDI), (c-d) patient interval (PI) and (e-f) diagnostic interval (DI) in weeks by ethnicity and IMD. *Dashed lines represent the group median (PI 1.1 weeks, DI 1.7 weeks, TDI 4.6 weeks); solid lines represent 26 weeks, respectively.*
