# Supplementary material for: Quantifying diagnostic intervals and routes to diagnosis for children and young people with cancer in the UK (Childhood Cancer Diagnosis study, CCD): a population-based observational study
Source: Lancet Reg Health Eur. 2025 May 27;54:101329. doi: 10.1016/j.lanepe.2025.101329 (PMC12266182; doi:10.1016/j.lanepe.2025.101329)
Supplement: Supplementary Figure S5 [file mmc5.pdf]

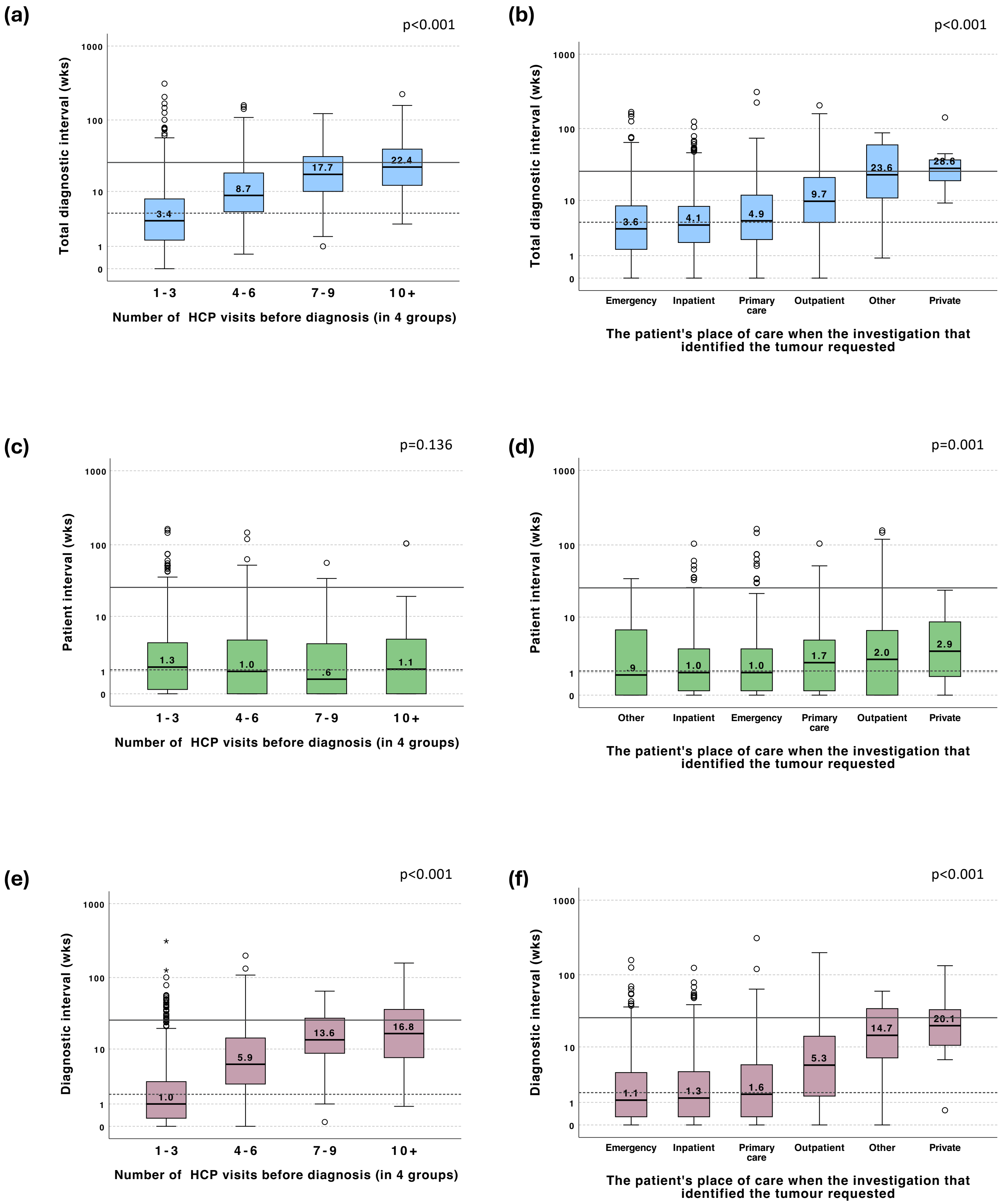

Figure S5: Box plots showing (a-b) total diagnostic interval (TDI), (c-d) patient interval (PI) and (e-f) diagnostic interval (DI) by number HCP visits before diagnosis and place of care where investigation leading to diagnosis was undertaken. *Dashed lines represent the group median (TDI 4.6 weeks, PI 1.1 weeks, DI 1.7 weeks); solid line represents 26 weeks, respectively.*
