# Supplementary material for: Quantifying diagnostic intervals and routes to diagnosis for children and young people with cancer in the UK (Childhood Cancer Diagnosis study, CCD): a population-based observational study
Source: Lancet Reg Health Eur. 2025 May 27;54:101329. doi: 10.1016/j.lanepe.2025.101329 (PMC12266182; doi:10.1016/j.lanepe.2025.101329)
Supplement: Supplementary Table S1 [file mmc7.pdf]

Table S1 Criteria for participant inclusion and exclusion

Inclusion criteria	Children and young people at age 0-18 years<br>**AND**<br>A new diagnosis of a childhood cancer<br>**WITH**<br>— the ability for their parent/guardian to give informed consent if age of the child is less than 16 years of age<br>— **Or** the ability for the young person to give informed consent if 16-18 years of age<br>— **Or** a consultee/legal representative is available to provide an opinion/consent if the young person is aged 16-18 and is deemed to lack capacity to consent for themselves.
Exclusion criteria	Age at diagnosis over 18 years of age<br>Patient diagnosed with cancer outside the UK
