# Supplementary material for: Quantifying diagnostic intervals and routes to diagnosis for children and young people with cancer in the UK (Childhood Cancer Diagnosis study, CCD): a population-based observational study
Source: Lancet Reg Health Eur. 2025 May 27;54:101329. doi: 10.1016/j.lanepe.2025.101329 (PMC12266182; doi:10.1016/j.lanepe.2025.101329)
Supplement: Supplementary Table S2 [file mmc8.pdf]

Table S2 Case report form and definition of key variables

THE CHILDHOOD CANCER DIAGNOSIS STUDY

Gender: ☐ Male ☐ Female

Ethnicity: ______________________________

Age: ______years ______ months

Year of birth (YYYY): ______________________

Diagnosis: ______________________

Tumour location: __________________

Laterality (if applicable): L / R / Midline/ Bilateral

Tumour stage: ____________________

Clinical risk group (if applicable): ____________

Key dates (DD/MM/YYYY)

- Date of symptom onset: ____________________________ ☐ Not known
- Date of first presentation to healthcare: _________________ ☐ Not known
- Date of clinical diagnosis: ____________________________ ☐ Not known
- Date of imaging: ___________________________________ ☐ Not known
- Date of biopsy/surgery: _____________________________ ☐ Not known

Route to diagnosis

- Who was the first healthcare professional (HCP) they saw about these symptoms:

  ☐ GP ☐ Paediatric emergency doctor ☐ Paediatrician ☐ Dentist ☐ Pharmacist

  ☐ Optometrist ☐Nurse practitioner ☐ Health visitor ☐ School nurse

  ☐ Other (please specify _________________________)
- How many HCP visits before diagnosis? _________ or ☐ 1-3 ☐ 4-6 ☐ 7-9 ☐10+
- Patient's place of care when the investigation that identified the tumour was requested:

  ☐ Primary care ☐ Outpatient ☐ Inpatient ☐ A&E ☐ Other _________________
- Was this an incidental finding?

  ☐ No ☐ Yes - asymptomatic ☐ Yes -with non-specific symptoms
- What was the source of referral leading to diagnosis?

Emergency presentation (A&E)	☐ Self-referral ☐ GP referral ☐ Optician referral ☐ Dentist referral<br>☐ MIU/Walk In Centre/NHS 111 ☐ Emergency transfer from another hospital<br>☐ Other HCP (please specify) ____________________________
GP referral	☐ Two week wait ☐ Routine referral ☐ Urgent referral to general paediatrician<br>☐ Other _____________________________________
Other	☐ Active surveillance (please specify ____________________________)<br>☐ Diagnosed by another specialty (e.g. ENT) ☐ Other _______________

Symptoms at diagnosis (Please tick all that apply)

Head, face, throat and neck

- ☐ Headache
- ☐ Vomiting
- ☐ Seizures
- ☐ Fits
- ☐ Visual abnormalities
- ☐ Papilloedema
- ☐ Leukocoria
- ☐ Abnormal eye movements
- ☐ Hearing loss
- ☐ Earache
- ☐ Torticollis/head tilt/stiff neck
- ☐ Sore throat/hoarse voice
- ☐ Difficulty swallowing
- ☐ Swollen glands
- ☐ Lump/swelling in face, jaw and skull
- ☐ Limited mouth opening
- ☐ Abnormal facial movements

Chest and Abdomen

- ☐ Shortness of breath
- ☐ Lump/swelling in chest wall or armpits
- ☐ Chest wall pain/axillary pain
- ☐ Abdominal pain/discomfort
- ☐ Abdominal distention/mass
- ☐ Haematuria
- ☐ Blood in stool
- ☐ Change in bowel habit
- ☐ Difficulty passing urine

Any other symptom not listed above:

Bones and Joints

- ☐ Bone/joint swelling
- ☐ Bone/joint pain
- ☐ Limp or leg weakness
- ☐ Slow in recovery after injury to bone/joint

Growth and Development

- ☐ Developmental delay
- ☐ Deterioration in balance/walking/speech
- ☐ Slow growth
- ☐ Weight loss
- ☐ Loss of appetite
- ☐ Early or late puberty
- ☐ Lump/swelling in pelvis, testicle or breast
- ☐ Unexplained bleeding after sex or between periods

Other symptoms

- ☐ Pallor
- ☐ Changes to moles
- ☐ Excessive bleeding/bruising/petechiae
- ☐ Persistent/recurrent unexplained screaming in young children
- ☐ Multiple infections
- ☐ Tiredness or fatigue
- ☐ Fever
- ☐ Night sweat

Key dates

Date of symptom onset

Date of first symptom is defined as the time point when first bodily changes and/or symptoms are noticed. Please record date of symptom onset as reported by the patient/parents/carers

Date of first presentation to healthcare

First presentation to healthcare with symptoms attributable to tumour (healthcare includes all doctors e.g. GPs/paediatricians, and community optometrist/opticians)

Date of diagnosis

Date of diagnosis: the first date of diagnosis whether clinically or histologically established. Please record date of clinical diagnosis, date of imaging and date of biopsy, either from pathology report or MDT meeting.

Route to diagnosis

The first healthcare professional

Healthcare professionals include all direct care practitioners and allied health professionals. For example: GP, paediatric emergency doctor, paediatrician, dentist, pharmacist, optometrist, nurse practitioner, health visitor, and school nurse.

Number of HCPs visits before diagnosis

Healthcare professional is defined as all direct care practitioners and allied health professionals. For example: GP, paediatric emergency doctor, paediatrician, other specialist (e.g., ENT), dentist, nurse practitioner, pharmacist, optometrist, orthoptist, audiologist, health visitor, school nurse.

Patient's place of care when the investigation that identified the tumour was requested:

Primary care, outpatient, inpatient, emergency department, other (please specify) or not known

Was this an incidental finding?

Incidental findings are defined as the previously undiagnosed cancer that are discovered unintentionally and may be unrelated to the medical condition which is being investigated or for which tests were being performed.

What was the source of referral leading to diagnosis?

This is to identify the source of referral which leading to the diagnosis.

- **Emergency presentation**[1]: An emergency route via A&E, emergency GP referral, emergency consultant outpatient referral, emergency transfer, emergency admission or attendance
- **GP referral**: This includes Two Week Wait (urgent GP referrals with a suspicion of cancer), as well as routine and urgent referrals where the patient was not referred under the Two Week Wait referral route.
- **Active surveillance**: patients under active surveillance (e.g., neurofibromatosis 1) and cancer was discovered during routine/follow up assessment.

[1] Routes to Diagnosis - NCIN Data Briefing http://www.ncin.org.uk/publications/data_briefings/routes_to_diagnosis

Multiple deprivation

The Index of Multiple Deprivation (IMD) is the official measure of relative deprivation for small areas. Each country in the UK has its own Index of Multiple Deprivation. Please use the lookup tools below to find out the name of the small area/data zone, deprivation rank, and decile for each participant.

- English Indices of Multiple Deprivation 2019
  https://imd-by-postcode.opendatacommunities.org/imd/2019
- The Welsh Index of Multiple Deprivation (WIND) 2019
  https://statswales.gov.wales/Catalogue/Community-Safety-and-Social-Inclusion/Welsh-Index-of-Multiple-Deprivation
- The Scottish Index of Multiple Deprivation 2020 (SIMD) v2
  https://www.gov.scot/publications/scottish-index-of-multiple-deprivation-2020v2-postcode-look-up
- Northern Ireland Multiple Deprivation Measure (NISRA) 2017
  https://deprivation.nisra.gov.uk
