# Supplementary material for: Quantifying diagnostic intervals and routes to diagnosis for children and young people with cancer in the UK (Childhood Cancer Diagnosis study, CCD): a population-based observational study
Source: Lancet Reg Health Eur. 2025 May 27;54:101329. doi: 10.1016/j.lanepe.2025.101329 (PMC12266182; doi:10.1016/j.lanepe.2025.101329)
Supplement: Supplementary Table S3 [file mmc9.pdf]

Table S3: Incident and recruited cases by Principal Treatment Centre

Principal Treatment Centre (PTC)	Total number of incident cases during study period	Total number of cases recruited to study	Percentage recruitment (%)	Pause in recruitment due to COVID
Aberdeen	51	35	69%	N
Belfast	76	51	67%	N
Birmingham	463	69	15%	N
Bristol	313	146	47%	N
Cambridge (0-16)	253	206	81%	N
Cardiff	49	5	10%	Y
Edinburgh	72	71	99%	N
Glasgow	158	105	66%	N
GOSH	325	207	64%	N
Leeds	261	109	42%	N
Leicester	59	51	86%	N
Liverpool	75	68	91%	Y
Manchester	333	202	61%	Y
Newcastle	93	87	94%	N
Nottingham	209	78	37%	Y
Oxford	97	97	100%	N
RMH	521	78	15%	N
Sheffield	140	116	83%	N
Southampton	130	127	98%	N
UCLH	U*	49	U	U

*U= unknown
