# Supplementary material for: Quantifying diagnostic intervals and routes to diagnosis for children and young people with cancer in the UK (Childhood Cancer Diagnosis study, CCD): a population-based observational study
Source: Lancet Reg Health Eur. 2025 May 27;54:101329. doi: 10.1016/j.lanepe.2025.101329 (PMC12266182; doi:10.1016/j.lanepe.2025.101329)
Supplement: Supplementary Table S4 [file mmc10.pdf]

Table S4 Total diagnostic interval, patient interval and diagnostic intervals in weeks

	**Total diagnostic interval (wks)** (valid n=1875)	**Patient interval (wks)** (valid n=1799)	**Diagnostic interval (wks)** (valid n=1853)
**Mean (SD)**	10.6 (19.3)	4.1 (11.1)	6.5 (15.8)
**Median (IQR)**	4.6 (2.0-11.4)	1.1 (0.1-4)	1.7 (0.4-5.9)
**Range (min, MAX)**	(0, 310.7)	0, 164	(0, 310.7)
**Intervals in 5 groups (n, %)**			
<= 4 weeks	831 (44)	1370 (76)	1264 (68)
4-12 weeks	600 (32)	291 (16)	348 (19)
12-26 weeks	262 (14)	88 (5)	131 (7)
26-52 weeks	124 (7)	31 (2)	79 (4)
> 52 weeks	58 (3)	19 (1)	31 (2)
