# Supplementary material for: Quantifying diagnostic intervals and routes to diagnosis for children and young people with cancer in the UK (Childhood Cancer Diagnosis study, CCD): a population-based observational study
Source: Lancet Reg Health Eur. 2025 May 27;54:101329. doi: 10.1016/j.lanepe.2025.101329 (PMC12266182; doi:10.1016/j.lanepe.2025.101329)
Supplement: Supplementary Table S5 [file mmc11.pdf]

Table S5 Diagnostic intervals by age, sex, ethnicity, IMD, region and diagnosis

		Total diagnostic interval (wks)			Patient interval (wks)			Diagnostic interval (wks)		
	n	Mean (SD)	Median (IQR)	(min, MAX)	Mean (SD)	Median (IQR)	(min, MAX)	Mean (SD)	Median (IQR)	(min, MAX)
**Age group**										
Under 1	152	6.4 (8.4)	3.7 (1.0-8.1)	(0, 51.0)	1.8 (4.5)	0.3 (0.0-1.2)	(0, 36.6)	4.5 (7.2)	1.6 (0.4-5.1)	(0, 37.4)
1-4	717	8.5 (17.7)	3.9 (1.6-7.9)	(0, 310.7)	3.0 (7.3)	0.7 (0.0-3.0)	(0, 104.0)	5.3 (16.3)	1.3 (0.3-4.1)	(0, 310.7)
5-9	432	11.3 (21.4)	4.6 (2.1-10.7)	(0, 205.4)	4.1 (13.2)	1.0 (0.0-3.7)	(0, 164.0)	7.0 (15.7)	1.7 (0.3-6.0)	(0, 157.6)
10-14	409	12.3 (20.9)	6.1 (2.6-13.7)	(0, 224.0)	6.0 (14.3)	2.0 (0.3-5.9)	(0, 146.6)	6.4 (13.4)	2.1 (0.4-5.9)	(0, 125.1)
15+	247	15.0 (20.3)	8.7 (3.0-17.4)	(0.1, 150.3)	5.9 (12.1)	2.6 (0.4-6.3)	(0, 119.4)	10.3 (20.8)	3.4 (0.8-11.4)	(0, 198.4)
**Sex**										
Male	1075	10.3 (19.2)	4.4 (2.0-10.9)	(0, 310.7)	4.0 (10.7)	1.0 (0.1-3.4)	(0, 164.0)	6.3 (16.6)	1.7 (0.3-5.3)	(0, 310.7)
Female	882	11.0 (19.3)	4.9 (2.1-12.3)	(0, 224.0)	4.3 (11.5)	1.3 (0.1-4.1)	(0, 146.6)	6.7 (14.8)	1.9 (0.4-6.4)	(0, 148.3)
**Ethnicity**										
White	1528	10.5 (17.1)	4.7 (2.0-12.0)	(0, 224.0)	3.9 (8.9)	1.0 (0.1-4.0)	(0, 119.4)	6.5 (14.0)	1.7 (0.4-6.0)	(0, 157.6)
Mixed	91	15.4 (41.2)	4.5 (2.1-12.4)	(0, 310.7)	5.5 (18.0)	1.4 (0.0-4.6)	(0, 156.6)	10.0 (35.8)	1.6 (0.3-4.4)	(0, 310.7)
Asian	156	10.1 (21.3)	4.4 (2.3-9.0)	(0.1, 167.3)	4.9 (18.1)	1.7 (0.1-3.7)	(0, 164.0)	5.3 (11.3)	2.0 (0.6-4.6)	(0, 108.1)
Black	43	11.8 (20.8)	4.3 (1.4-12.9)	(0.1, 104.9)	6.1 (17.7)	1.2 (0.4-3.5)	(0, 104.0)	10.2 (32.2)	0.9 (0.3-4.3)	(0, 198.4)
Other ethnic group	56	9.5 (20.6)	4.1 (2.1-9.4)	(0.1, 146.1)	4.6 (19.8)	0.7 (0.0-2.0)	(0, 145.4)	5.2 (8.4)	2.1 (0.7-6.6)	(0, 50.6)
Not known	83	8.1 (11.5)	4.5 (2.1-8.7)	(0, 65.3)	3.5 (6.0)	1.4 (0.3-4.4)	(0, 36.1)	3.9 (8.0)	1.6 (0.4-3.4)	(0, 56.9)
**IMD in quintile**										
1 Most deprived	391	11.5 (25.2)	4.4 (2.1-12.3)	(0, 310.7)	4.1 (11.2)	0.9 (0.0-3.9)	(0, 145.4)	7.2 (21.5)	1.7 (0.3-5.9)	(0, 310.7)
2	341	8.8 (12.3)	4.4 (1.9-9.9)	(0.1, 103.0)	3.2 (6.9)	1.0 (0.0-3.4)	(0, 63.6)	5.4 (10.2)	1.4 (0.3-5.8)	(0, 103.0)
3	341	10.6 (18.8)	4.6 (2.1-11.0)	(0, 158.6)	4.6 (11.6)	1.5 (0.1-4.2)	(0, 146.6)	6.0 (15.1)	1.3 (0.3-4.6)	(0, 157.6)
4	392	10.1 (17.4)	4.4 (2.1-11.1)	(0, 205.4)	4.0 (11.7)	1.1 (0.0-3.5)	(0, 156.6)	6.6 (15.0)	2.0 (0.4-5.9)	(0, 198.4)
5 Least deprived	369	11.7 (19.7)	4.8 (2.0-12.9)	(0, 167.3)	4.3 (11.7)	1.3 (0.1-3.5)	(0, 164.0)	7.2 (15.5)	1.9 (0.3-7.0)	(0, 132.6)
**Region**										
England & Wales	1695	11.0 (20.1)	4.6 (2.1-12.0)	(0, 310.7)	4.2 (11.7)	1.0 (0.1-4.0)	(0, 164.0)	6.8 (16.4)	1.9 (0.4-6.0)	(0, 310.7)
Scotland	211	8.0 (12.3)	4.2 (1.9-8.1)	(0, 100.6)	3.8 (5.9)	2.0 (0.3-4.4)	(0, 34.6)	4.9 (12.6)	1.0 (0.1-4.0)	(0, 106.9)
Northern Ireland	51	7.1 (9.4)	4.6 (1.7-8.6)	(0, 51.0)	2.5 (5.4)	1.0 (0.1-3.0)	(0, 36.6)	4.4 (7.1)	0.4 (0.0-6.0)	(0, 34.9)

	n	Total diagnostic interval (wks)			Patient interval (wks)			Diagnostic interval (wks)		
		Mean (SD)	Median (IQR)	(min, MAX)	Mean (SD)	Median (IQR)	(min, MAX)	Mean (SD)	Median (IQR)	(min, MAX)
**Diagnosis**										
Leukaemia	778	5.5 (7.6)	3.1 (1.4-6.1)	(0, 68.1)	2.5 (4.5)	1.0 (0.1-3.0)	(0, 52.7)	2.9 (6.6)	0.7 (0.1-2.7)	(0, 68.1)
Lymphoma & related	254	13.3 (15.7)	7.9 (3.6-16.4)	(0.1, 104.4)	4.7 (8.4)	1.9 (0.1-5.9)	(0, 52.1)	8.4 (13.0)	3.1 (1.3-10.4)	(0, 103.0)
CNS tumour	275	18.6 (33.2)	7.6 (3.0-19.0)	(0, 310.7)	7.3 (16.8)	2.0 (0.2-5.2)	(0, 145.4)	11.7 (28.8)	2.5 (0.6-9.5)	(0, 310.7)
Neuroblastoma	105	6.6 (7.7)	4.4 (1.6-7.7)	(0, 49.3)	2.0 (4.5)	0.4 (0.0-2.3)	(0, 35.9)	4.5 (7.0)	1.6 (0.7-5.4)	(0, 49.3)
Retinoblastoma	32	11.0 (14.9)	4.1 (1.4-14.6)	(0, 50.4)	5.8 (10.6)	0.9 (0.1-5.4)	(0, 43.0)	5.6 (12.8)	0.8 (0.1-3.3)	(0, 50.4)
Renal tumour	139	5.7 (9.8)	2.3 (0.9-5.0)	(0, 58.0)	2.1 (5.4)	0.3 (0.0-1.7)	(0, 46.7)	3.3 (7.1)	0.9 (0.3-3.0)	(0, 58.0)
Hepatic tumour	40	8.3 (10.2)	5.1 (1.9-8.0)	(0, 42.7)	3.8 (7.5)	1.0 (0.0-4.3)	(0, 39.4)	4.6 (7.0)	1.6 (0.7-5.6)	(0, 36.6)
Bone tumour	124	19.7 (24.7)	12.6 (6.6-23.4)	(0, 167.3)	8.4 (18.2)	3.1 (0.3-8.7)	(0, 164.0)	11.1 (19.2)	4.6 (1.9-12.0)	(0, 157.6)
Soft tissue sarcoma	128	13.4 (25.0)	6.9 (3.1-13.6)	(0.1, 205.4)	5.4 (20.3)	1.0 (0.0-3.1)	(0, 156.6)	9.2 (20.0)	4.3 (1.6-8.6)	(0, 198.4)
Germ cell tumour	28	12.7 (23.6)	5.9 (2.4-14.0)	(0.7, 125.1)	3.4 (5.1)	0.5 (0.0-4.4)	(0, 18.7)	9.2 (23.8)	2.7 (0.6-9.3)	(0, 125.1)
Carcinoma & melanoma	13	31.7 (48.4)	9.6 (4.9-25.6)	(1.9, 150.3)	11.5 (34.1)	0.4 (0.0-3.6)	(0, 119.4)	22.7 (34.3)	7.7 (3.6-26.6)	(1.9, 123.9)
Other & unspecified malignant	5	20.3 (13.8)	16.4 (15.1-33.4)	(1.9, 34.7)	2.4 (2.3)	3.3 (0.0-4.4)	(0, 4.4)	17.9 (13.6)	12.0 (11.9-29.0)	(1.9, 34.7)
LCH*	36	20.4 (24.2)	8.8 (5.1-27.6)	(1.0, 88.4)	3.9 (10.0)	0.9 (0.0-2.0)	(0, 52.1)	17.1 (21.1)	7.8 (3.4-27.6)	(0.7, 82.3)

*Langerhans Cell histiocytosis (LCH) and other histiocytosis
