# Supplementary material for: Quantifying diagnostic intervals and routes to diagnosis for children and young people with cancer in the UK (Childhood Cancer Diagnosis study, CCD): a population-based observational study
Source: Lancet Reg Health Eur. 2025 May 27;54:101329. doi: 10.1016/j.lanepe.2025.101329 (PMC12266182; doi:10.1016/j.lanepe.2025.101329)
Supplement: Supplementary Table S6 [file mmc12.pdf]

Table S6 Diagnostic intervals by route to diagnosis

	n	Total diagnostic interval (wks)			Patient interval (wks)			Diagnostic interval (wks)		
		Mean (SD)	Median (IQR)*	(min, MAX)	Mean (SD)	Median (IQR)*	(min, MAX)	Mean (SD)	Median (IQR)*	(min, MAX)
**The first HCP seen**										
GP	1113	11.5 (19.1)	5.4 (2.6-13.0)	(0, 224.0)	4.1 (11.2)	1.4 (0.1-4.1)	(0, 164.0)	7.6 (16.0)	2.4 (0.6-7.4)	(0, 198.4)
Emergency doctor	562	7.1 (13.0)	3.1 (1.3-7.7)	(0, 146.1)	3.3 (9.8)	0.8 (0.0-2.9)	(0, 145.4)	3.8 (9.3)	1.0 (0.3-3.1)	(0, 125.1)
Paediatrician	107	11.6 (20.8)	4.9 (2.3-9.1)	(0, 150.3)	6.4 (15.3)	2.0 (0.0-4.3)	(0, 119.4)	5.4 (10.6)	1.0 (0.1-5.0)	(0, 57.6)
Sub-specialist doctor	35	22.8 (58.1)	5.9 (1.9-13.0)	(0.4, 310.7)	6.4 (10.4)	1.9 (0.0-8.4)	(0, 43.0)	15.1 (55.9)	1.7 (0.3-4.1)	(0, 310.7)
Optometrist	35	18.2 (26.0)	5.8 (2.4-25.7)	(0.1, 122.0)	11.2 (17.8)	4.0 (0.4-12.7)	(0, 74.6)	7.3 (14.1)	1.8 (0.4-7.0)	(0, 65.3)
WIC/ UCC/ MIU	16	5.5 (6.2)	3.6 (1.8-6.3)	(0.3, 23.1)	2.3 (5.4)	0.7 (0.3-1.9)	(0, 21.7)	3.4 (4.3)	2.1 (0.9-3.7)	(0, 16.9)
NHS111/ NHS24	14	2.1 (1.7)	1.5 (0.7-3.0)	(0.1, 5.6)	1.0 (1.1)	0.6 (0.1-1.4)	(0, 4.0)	1.1 (1.5)	0.6 (0.1-1.1)	(0.1, 5.3)
Nurse practitioner	13	16.6 (23.1)	6.6 (2.0-14.7)	(0.6, 69.4)	5.5 (11.3)	0.9 (0.1-3.0)	(0, 39.1)	11.2 (17.9)	2.4 (2.0-11.3)	(0.3, 64.0)
Pre/peri-natal	12	3.0 (3.5)	2.0 (0.0-6.0)	(0, 10.6)	0.0 (0.1)	0.0 (0.0-0.0)	(0, 0.3)	3.0 (3.4)	2.4 (0.1-4.9)	(0, 10.6)
Health visitor	10	23.6 (19.4)	19.6 (10.6-37.6)	(1.0, 51.0)	7.1 (13.3)	0.3 (0.0-6.6)	(0, 36.6)	29.8 (35.5)	13.8 (7.5-43.9)	(0.7, 106.9)
Dentist	10	6.4 (10.2)	3.0 (2.0-4.7)	(1.0, 35.0)	3.0 (6.6)	0.7 (0.1-2.3)	(0, 21.6)	3.4 (4.1)	1.9 (0.9-4.4)	(0, 13.4)
Physiotherapist	6	30.7 (33.0)	13.3 (11.7-31.9)	(9.1, 87.4)	4.3 (0.0)	4.3 (4.3-4.3)	(4.3, 4.3)	14.6 (14.7)	7.4 (4.9-31.6)	(4.9, 31.6)
Private	5	12.6 (18.4)	4.4 (4.3-4.9)	(4.0, 45.4)	3.4 (1.6)	4.0 (3.9-4.1)	(0.6, 4.3)	9.2 (19.9)	0.1 (0.1-0.9)	(0.1, 44.9)
Pharmacist	3	1.3 (1.1)	1.4 (-)	(0.1, 2.4)	0.2 (0.3)	0.0 (-)	(0, 0.6)	1.1 (1.2)	0.9 (-)	(0.1, 2.4)
School nurse	2	11.1 (12.9)	11.1 (-)	(2.0, 20.3)	4.1 (3.0)	4.1 (-)	(2.0, 6.3)	7.0 (9.9)	7.0 (-)	(0, 14.0)
Other	8	18.1 (13.5)	17.9 (6.9-28.8)	(0.9, 36.6)	4.8 (7.6)	0.9 (0.0-8.7)	(0, 20.6)	15.1 (13.5)	9.1 (5.3-27.6)	(0, 36.6)
Not known	5	40.6 (47.7)	27.0 (4.1-77.1)	(4.0, 104.4)	19.6 (27.8)	19.6 (0.0-39.3)	(0, 39.3)	23.5 (36.1)	4.0 (1.4-65.1)	(1.4, 65.1)
**Number of HCP visits before diagnosis**										
1-3	1438	7.4 (15.5)	3.4 (1.4-7.7)	(0, 310.7)	3.9 (10.2)	1.3 (0.1-3.9)	(0, 164.0)	3.5 (11.5)	1.0 (0.3-3.0)	(0, 310.7)
4-6	375	16.0 (20.9)	8.7 (4.9-18.6)	(0.6, 158.6)	4.7 (13.0)	1.0 (0.0-4.3)	(0, 146.6)	12.1 (19.0)	5.9 (2.7-14.6)	(0, 198.4)
7-9	88	22.8 (19.1)	17.7 (10.0-31.4)	(1.0, 122.0)	3.8 (8.5)	0.6 (0.0-3.7)	(0, 56.7)	19.3 (16.0)	13.6 (8.6-27.6)	(0.1, 65.3)
10+	51	39.3 (45.3)	22.4 (12.3-39.9)	(3.0, 224.0)	7.8 (21.6)	1.1 (0.0-4.4)	(0, 104.3)	31.2 (39.1)	16.8 (7.4-36.6)	(0.9, 157.6)

	n	Total diagnostic interval (wks)			Patient interval (wks)			Diagnostic interval (wks)		
		Mean (SD)	Median (IQR)*	(min, MAX)	Mean (SD)	Median (IQR)*	(min, MAX)	Mean (SD)	Median (IQR)*	(min, MAX)
**The patient's place of care when the investigation that identified the tumour was requested**										
Inpatient	784	7.4 (11.1)	4.1 (2.0-8.1)	(0, 123.9)	2.9 (6.7)	1.0 (0.1-3.1)	(0, 104.0)	4.3 (9.3)	1.3 (0.3-4.1)	(0, 123.9)
Emergency	572	8.6 (16.5)	3.6 (1.4-8.3)	(0, 167.3)	3.8 (12.0)	1.0 (0.1-3.1)	(0, 164.0)	4.7 (11.8)	1.1 (0.3-4.0)	(0, 157.6)
Outpatient	366	18.3 (25.2)	9.7 (4.6-21.3)	(0, 205.4)	6.7 (16.1)	2.0 (0.0-6.3)	(0, 156.6)	12.7 (21.6)	5.3 (1.4-14.4)	(0, 198.4)
Primary care	208	12.4 (29.7)	4.9 (2.3-11.9)	(0, 310.7)	5.1 (11.2)	1.7 (0.1-4.4)	(0, 104.3)	7.0 (25.0)	1.6 (0.3-5.4)	(0, 310.7)
Private	11	36.7 (36.5)	28.6 (14.9-39.9)	(9.1, 141.1)	6.8 (8.5)	2.9 (0.6-8.6)	(0, 24.1)	29.9 (36.7)	20.1 (9.3-38.9)	(0.6, 132.6)
Other	10	34.1 (30.3)	23.6 (10.9-60.0)	(0.9, 87.4)	5.7 (11.3)	0.9 (0.0-6.4)	(0, 34.9)	22.5 (20.0)	14.7 (6.9-34.7)	(0, 60.0)
Not known	5	11.3 (21.5)	2.1 (0.6-4.0)	(0.3, 49.7)	0.6 (0.8)	0.3 (0.1-1.1)	(0, 1.9)	1.1 (1.9)	0.3 (0.1-2.1)	(0, 4.0)
**Source of referral leading to diagnosis**										
Emergency presentation	1312	8.8 (16.1)	4.1 (1.9-9.0)	(0, 224.0)	3.8 (11.0)	1.0 (0.1-3.6)	(0, 164.0)	4.8 (10.7)	1.4 (0.3-4.3)	(0, 157.6)
GP referral	463	11.4 (16.5)	5.9 (2.4-13.6)	(0, 158.6)	4.2 (9.7)	1.7 (0.1-4.3)	(0, 146.6)	8.0 (17.0)	2.3 (0.4-7.9)	(0, 198.4)
Other	178	23.0 (37.1)	10.4 (3.9-27.1)	(0, 310.7)	6.5 (14.7)	1.0 (0.0-5.1)	(0, 119.4)	15.7 (33.0)	4.9 (0.9-14.9)	(0, 310.7)
Unknown	3	2.8 (1.2)	2.3 (-)	(1.9, 4.1)	2.6 (1.2)	2.0 (-)	(1.7, 4.0)	0.2 (0.1)	0.1 (-)	(0.1, 0.3)
**Incidental finding**										
No	1913	10.7 (19.4)	4.6 (2.0-11.6)	(0, 310.7)	4.2 (11.2)	1.1 (0.1-4.0)	(0, 164.0)	6.5 (11.5)	1.7 (0.4-5.7)	(0, 310.7)
Yes										
Asymptomatic	40	6.5 (7.7)	3.9 (1.9-8.3)	(0, 31.9)	1.2 (2.3)	0.0 (0.0-2.0)	(0, 8.9)	6.7 (11.7)	2.4 (0.1-7.6)	(0, 57.6)
Antenatal diagnosis	3	16.2 (8.0)	16.2 (-)	(10.6, 21.9)	0.1 (0.1)	0.1 (-)	(0, 0.1)	11.8 (9.4)	10.6 (-)	(3.0, 21.7)

*IQR is not reported when n ≤ 3. Please refer to the median, minimum, and maximum values for the data distribution of these subcategories.
