# Supplementary material for: Quantifying diagnostic intervals and routes to diagnosis for children and young people with cancer in the UK (Childhood Cancer Diagnosis study, CCD): a population-based observational study
Source: Lancet Reg Health Eur. 2025 May 27;54:101329. doi: 10.1016/j.lanepe.2025.101329 (PMC12266182; doi:10.1016/j.lanepe.2025.101329)
Supplement: Supplementary Table S7 [file mmc13.pdf]

Table S7 Factors associated with lengthy total diagnostic interval

		Total Diagnostic interval Q4: 11.5+ wks^			
	Total	Q1-3 (n=1406)	Q4 (n=469)	Crude OR	Adj OR*
	n (Col%)	n (Col%)	n (Col%)	(95%CI)	(95%CI)
**Age group**				*p<0.001*	*p<0.001*
Under 1	142 (8%)	118 (8%)	24 (5%)	1.00	1.00
1-4	685 (37%)	563 (40%)	122 (26%)	1.07 (0.66-1.72)	1.22 (0.73-2.06)
5-9	414 (22%)	314 (22%)	100 (21%)	1.57 (0.96-2.56)	1.73 (0.99-3.01)
10-14	395 (21%)	276 (20%)	119 (25%)	2.12 (1.30-3.46)	1.89 (1.07-3.32)
15+	239 (13%)	135 (10%)	104 (22%)	3.79 (2.28-6.30)	3.58 (1.95-6.57)
**Sex**				*p=0.094*	*p=0.151*
Male	1034 (55%)	791 (56%)	243 (52%)	1.00	1.00
Female	841 (45%)	615 (44%)	226 (48%)	1.20 (0.97-1.48)	1.19 (0.94-1.52)
**Ethnicity**				*p=0.244*	*p=0.733*
White	1462 (81%)	1081 (81%)	381 (83%)	1.00	1.00
Other ethnic group	335 (19%)	258 (19%)	77 (17%)	0.85 (0.64-1.12)	0.95 (0.69-1.29)
**IMD in quintile**				*p=0.163*	*p=0.170*
1 Most deprived	374 (21%)	278 (21%)	96 (22%)	1.00	1.00
2	329 (19%)	259 (20%)	70 (16%)	0.78 (0.55-1.11)	0.70 (0.48-1.04)
3	319 (18%)	244 (19%)	75 (17%)	0.89 (0.63-1.26)	0.84 (0.58-1.24)
4	379 (22%)	286 (22%)	93 (21%)	0.94 (0.68-1.31)	0.90 (0.62-1.29)
5 Least deprived	354 (20%)	250 (19%)	104 (24%)	1.20 (0.87-1.67)	1.12 (0.78-1.61)
**Diagnosis**				*p<0.001*	*p<0.001*
Leukaemia	749 (40%)	659 (47%)	90 (19%)	1.00	1.00
Lymphoma & related	244 (13%)	153 (11%)	91 (19%)	4.36 (3.10-6.12)	3.45 (2.34-5.10)
CNS tumour	263 (14%)	162 (12%)	101 (22%)	4.57 (3.28-6.36)	5.24 (3.69-7.46)
Neuroblastoma	99 (5%)	81 (6%)	18 (4%)	1.63 (0.93-2.84)	2.34 (1.30-4.22)
Retinoblastoma	29 (2%)	21 (1%)	8 (2%)	2.79 (1.20-6.48)	3.81 (1.52-9.59)
Renal tumour	131 (7%)	114 (8%)	17 (4%)	1.09 (0.63-1.90)	1.36 (0.76-2.45)
Hepatic tumour	38 (2%)	31 (2%)	7 (1%)	1.65 (0.71-3.87)	2.22 (0.92-5.35)
Bone tumour	119 (6%)	56 (4%)	63 (13%)	8.24 (5.40-12.56)	6.36 (3.99-10.12)
Soft tissue sarcoma	124 (7%)	84 (6%)	40 (9%)	3.49 (2.25-5.39)	3.79 (2.38-6.04)
Germ cell tumour	27 (1%)	17 (1%)	10 (2%)	4.31 (1.91-9.70)	3.99 (1.54-10.38)
Carcinoma & melanoma	13 (0.7%)	7 (0.5%)	6 (1%)	6.28 (2.06-19.09)	4.36 (1.25-15.23)
Other & unspecified$	5 (0.3%)	1 (0.1%)	4 (0.9%)	29.29 (3.24-264.95)	--
LCH	34 (2%)	20 (1%)	14 (3%)	5.13 (2.50-10.51)	6.86 (3.20-14.69)

^ Patients with missing data were not included in the analysis, valid n=1875.

**Adjusted for all factors shown in the table*

$Less than 10 cases in the group
