# Supplementary material for: Quantifying diagnostic intervals and routes to diagnosis for children and young people with cancer in the UK (Childhood Cancer Diagnosis study, CCD): a population-based observational study
Source: Lancet Reg Health Eur. 2025 May 27;54:101329. doi: 10.1016/j.lanepe.2025.101329 (PMC12266182; doi:10.1016/j.lanepe.2025.101329)
Supplement: Supplementary Table S8 [file mmc14.pdf]

Table S8 Factors associated with TDI longer than 6 months (26 weeks)

	Total	Total diagnostic interval		Crude OR	Adj OR*
		<= 26 weeks (n=1693)	> 26 weeks (n=182)		
	n (Col%)	n (Col%)	n (Col%)	(95%CI)	(95%CI)
**Age group**				*p=0.003*	*p=0.079*
Under 1	142 (8%)	135 (8%)	7 (4%)	1.00	1.00
1-4	685 (37%)	635 (38%)	50 (27%)	1.52 (0.67-3.42)	1.80 (0.75-4.30)
5-9	414 (22%)	369 (22%)	45 (25%)	2.35 (1.04-5.34)	2.76 (1.11-6.86)
10-14	395 (21%)	350 (21%)	45 (25%)	2.48 (1.09-5.63)	2.48 (0.98-6.25)
15+	239 (13%)	204 (12%)	35 (19%)	3.31 (1.43-7.67)	3.33 (1.26-8.77)
**Sex**				*p=0.248*	*p=0.202*
Male	1034 (55%)	941 (56%)	93 (51%)	1.00	1.00
Female	841 (45%)	752 (44%)	89 (49%)	1.20 (0.88-1.63)	1.25 (0.89-1.75)
**Ethnicity**				*p=0.397*	*p=0.695*
White	1462 (81%)	1313 (81%)	149 (84%)	1.00	1.00
Other ethnic group	335 (19%)	306 (19%)	29 (16%)	0.84 (0.55-1.27)	0.91 (0.58-1.43)
**IMD in quintile**				*p=0.482*	*p=0.455*
1 Most deprived	374 (21%)	333 (21%)	41 (24%)	1.00	1.00
2	329 (19%)	302 (19%)	27 (16%)	0.73 (0.44-1.21)	0.64 (0.37-1.10)
3	319 (18%)	290 (18%)	29 (17%)	0.81 (0.49-1.34)	0.74 (0.43-1.27)
4	379 (22%)	346 (22%)	33 (19%)	0.77 (0.48-1.25)	0.69 (0.41-1.16)
5 Least deprived	354 (20%)	313 (20%)	41 (24%)	1.06 (0.67-1.68)	0.87 (0.53-1.44)
**Diagnosis main group**				*p<0.001*	*p<0.001*
Leukaemia	749 (40%)	726 (43%)	23 (13%)	1.00	1.00
Lymphoma & related	244 (13%)	209 (12%)	35 (19%)	5.29 (3.06-9.14)	3.92 (2.13-7.24)
CNS tumour	263 (14%)	212 (13%)	51 (28%)	7.59 (4.53-12.72)	7.85 (4.62-13.33)
Neuroblastoma	99 (5%)	96 (6%)	3 (2%)	0.99 (0.29-3.35)	1.28 (0.37-4.45)
Retinoblastoma	29 (2%)	23 (1%)	6 (3%)	8.23 (3.06-22.15)	10.27 (3.39-31.07)
Renal tumour	131 (7%)	125 (7%)	6 (3%)	1.52 (0.60-3.80)	1.47 (0.54-4.01)
Hepatic tumour	38 (2%)	35 (2%)	3 (2%)	2.71 (0.78-9.44)	3.55 (0.99-12.80)
Bone tumour	119 (6%)	94 (6%)	25 (14%)	8.40 (4.58-15.38)	7.09 (3.71-13.55)
Soft tissue sarcoma	124 (7%)	111 (7%)	13 (7%)	3.70 (1.82-7.51)	3.66 (1.74-7.67)
Germ cell tumour	27 (1%)	26 (2%)	1 (0.5%)	1.21 (0.16-9.34)	1.36 (0.17-10.76)
Carcinoma & melanoma	13 (0.7%)	10 (0.6%)	3 (2%)	9.47 (2.44-36.72)	4.96 (0.98-25.07)
Other & unspecified[$]	5 (0.3%)	3 (0.2%)	2 (1%)	21.04 (3.35-132.07)	70.84 (6.02-833.86)
LCH	34 (2%)	23 (1%)	11 (6%)	15.10 (6.58-34.61)	19.22 (8.01-46.10)

^ *Patients with missing data were not included in the analysis, valid n=1875.*

**Adjusted for all factors shown in the table*

$ *Less than 10 cases in the group*
