# Supplementary material for: Quantifying diagnostic intervals and routes to diagnosis for children and young people with cancer in the UK (Childhood Cancer Diagnosis study, CCD): a population-based observational study
Source: Lancet Reg Health Eur. 2025 May 27;54:101329. doi: 10.1016/j.lanepe.2025.101329 (PMC12266182; doi:10.1016/j.lanepe.2025.101329)
Supplement: Supplementary Table S9 [file mmc15.pdf]

Table S9 Factors associated with multiple visits (4 times or more) before diagnosis

	Total	Number of HCP visits before diagnosis^		Crude OR	Adj OR*
		1-3 visits (n=1438)	4+ visits (n=514)		
	n (Col%)	n (Col%)	n (Col%)	(95%CI)	(95%CI)
**Age group**				*p=0.278*	*p=0.690*
Under 1	151 (8%)	116 (8%)	35 (7%)	1.00	1.00
1-4	716 (37%)	536 (37%)	180 (35%)	1.11 (0.74-1.68)	1.28 (0.81-2.02)
5-9	431 (22%)	320 (22%)	111 (22%)	1.15 (0.74-1.78)	1.26 (0.77-2.08)
10-14	408 (21%)	298 (21%)	110 (21%)	1.22 (0.79-1.89)	1.11 (0.66-1.85)
15+	246 (13%)	168 (12%)	78 (15%)	1.54 (0.97-2.45)	1.35 (0.77-2.37)
**Sex**				*p=0.934*	*p=0.836*
Male	1074 (55%)	792 (55%)	282 (55%)	1.00	1.00
Female	878 (45%)	646 (45%)	232 (45%)	1.01 (0.82-1.24)	1.02 (0.82-1.28)
**Ethnicity**				*p=0.484*	*p=0.966*
White	1523 (81%)	1112 (81%)	411 (83%)	1.00	1.00
Other ethnic group	346 (19%)	259 (19%)	87 (17%)	0.91 (0.70-1.19)	0.99 (0.75-1.32)
**IMD in quintile**				*p=0.498*	*p=0.508*
1 Most deprived	390 (21%)	285 (21%)	105 (22%)	1.00	1.00
2	341 (19%)	261 (19%)	80 (17%)	0.83 (0.59-1.16)	0.91 (0.64-1.29)
3	341 (19%)	253 (19%)	88 (18%)	0.94 (0.68-1.31)	0.95 (0.67-1.35)
4	390 (21%)	276 (20%)	114 (24%)	1.12 (0.82-1.53)	1.19 (0.85-1.66)
5 Least deprived	368 (20%)	274 (20%)	94 (20%)	0.93 (0.67-1.29)	0.92 (0.65-1.30)
**Diagnosis main group**				*p<0.001*	*p<0.001*
Leukaemia	776 (40%)	628 (44%)	148 (29%)	1.00	1.00
Lymphoma & related	253 (13%)	174 (12%)	79 (15%)	1.93 (1.40-2.65)	1.97 (1.36-2.85)
CNS tumour	274 (14%)	173 (12%)	101 (20%)	2.48 (1.83-3.36)	2.69 (1.96-3.70)
Neuroblastoma	105 (5%)	76 (5%)	29 (6%)	1.62 (1.02-2.57)	1.73 (1.05-2.83)
Retinoblastoma	32 (2%)	28 (2%)	4 (0.8%)	0.61 (0.21-1.75)	0.75 (0.25-2.23)
Renal tumour	138 (7%)	119 (8%)	19 (4%)	0.68 (0.40-1.14)	0.72 (0.42-1.24)
Hepatic tumour	40 (2%)	31 (2%)	9 (2%)	1.23 (0.57-2.64)	1.38 (0.63-3.01)
Bone tumour	124 (6%)	82 (6%)	42 (8%)	2.17 (1.44-3.28)	2.58 (1.64-4.04)
Soft tissue sarcoma	128 (7%)	83 (6%)	45 (9%)	2.30 (1.53-3.45)	2.50 (1.63-3.84)
Germ cell tumour	28 (1%)	21 (1%)	7 (1%)	1.41 (0.59-3.39)	1.01 (0.33-3.08)
Carcinoma & melanoma	13 (0.7%)	5 (0.3%)	8 (2%)	6.79 (2.19-21.05)	4.98 (1.48-16.77)
Other & unspecified$	5 (0.3%)	2 (0.1%)	3 (0.6%)	6.36 (1.05-38.43)	8.59 (0.76-96.66)
LCH	36 (2%)	16 (1%)	20 (4%)	5.30 (2.68-10.48)	7.48 (3.54-15.82)

^ Patients with missing data were not included in the analysis, valid n=1952.
**Adjusted for all factors shown in the table*
$Less than 10 cases in the group
