# Supplementary material for: Quantifying diagnostic intervals and routes to diagnosis for children and young people with cancer in the UK (Childhood Cancer Diagnosis study, CCD): a population-based observational study
Source: Lancet Reg Health Eur. 2025 May 27;54:101329. doi: 10.1016/j.lanepe.2025.101329 (PMC12266182; doi:10.1016/j.lanepe.2025.101329)
Supplement: Supplementary Table S10 [file mmc16.pdf]

Table S10 Comparison of age, sex, country, and diagnosis between UK CTYA cancer cases and CCD cases (ages 0–14 years only; excluding LCH)

	CCD Study (0-14 years excl LCH) n=1,676		CTYA UK cancer 1997-2016 (0-14 yr) Mean no of cases per year, n=1,645		
	**n**	**Col%**	**Avg no of cases per year**	**Col%**	**p-value**
**Age group**					*0.004*
0-4	846	50%	745	*45%*	
5-9	425	25%	431	*26%*	
10-14	405	24%	470	*29%*	
**Sex**					*0.863*
Male	924	55%	902	*55%*	
Female	752	45%	743	*45%*	
**Country**					*0.001*
England & Wales	1,441	86%	1,463	*89%*	
Scotland	192	12%	129	*8%*	
Northern Ireland	43	3%	53	*3%*	
**Diagnosis**					*<0.001*
Leukaemia	709	42%	517	*31%*	
Lymphoma & related	169	10%	168	*10%*	
CNS tumour	255	15%	434	*26%*	
Neuroblastoma	105	6%	98	*6%*	
Retinoblastoma	32	2%	42	*3%*	
Renal tumour	139	8%	91	*6%*	
Hepatic tumour	38	2%	20	*1%*	
Bone tumour	90	5%	69	*4%*	
Soft tissue sarcoma	110	7%	105	*6%*	
Germ cell tumour	18	1%	36	*2%*	
Carcinoma & melanoma	8	0.5%	54	*3%*	
Other & unspecified malignant	3	0.2%	12	*1%*	
